# Structural insights into a flavin-dependent dehalogenase HadA explain catalysis and substrate inhibition *via* quadruple π-stacking

**DOI:** 10.1016/j.jbc.2021.100952

**Published:** 2021-07-10

**Authors:** Panu Pimviriyakul, Aritsara Jaruwat, Penchit Chitnumsub, Pimchai Chaiyen

**Affiliations:** 1 Department of Biochemistry, Faculty of Science, Kasetsart University, Bangkok, Thailand; 2 National Center for Genetic Engineering and Biotechnology, Pathumthani, Thailand; 3 School of Biomolecular Science and Engineering, Vidyasirimedhi Institute of Science and Technology (VISTEC), Rayong, Thailand

**Keywords:** flavin-dependent monooxygenase/dehalogenase, crystal structure, biodegradation, enzyme kinetics, site-directed mutagenesis, inhibition mechanism, quadruple π-stacking, halogenated phenol, nitrophenol, BQ, benzoquinone, C_2_, *p*-hydroxyphenylacetate hydroxylase from *Acinetobacter baumannii*, *Cn*TcpA, 2,4,6-trichlorophenol monooxygenase from *C. nantongensis* X1^T^, CphC-I, a two-component flavin monooxygenase from *Arthrobacter chlorophenolicus* A6, 4CP, 4-chlorophenol, DcmB1, two-component flavin monooxygenase from *Rhodococcus* sp. JT-3, *Ec*HpaB, *p*-hydroxyphenylacetate hydroxylase from *Escherichia coli*, FAD, oxidized form of flavin adenine dinucleotide, FADH^–^, reduced form of flavin adenine dinucleotide, Glc-6-P, glucose-6-phosphate, Glc-6-PD, glucose-6-phosphate dehydrogenase, HadA, a flavin monooxygenase component from *Raltonia pikettii*, HadX, a flavin reductase component from *Raltonia pikettii*, 4HPA, *p*-hydroxyphenylacetate, HPs, halogenated phenols, HnpA, FAD-dependent two-component monooxygenase from *Cupriavidus* sp. CNP-8, HQ, hydroquinone, NP, nitrophenol, 4NP, 4-nitrophenol, NpcA, 4-nitrophenol monooxygenase from *Rhodococcus opacus* SAO101, NpdA2, *p*-nitrophenol monooxygenase from *Arthrobacter sp.* Strain JS443, NpsA1, *p*-nitrophenol monooxygenase from *Rhodococcus sp.* strain PN1, PcpB, pentachlorophenol hydroxylase from *Sphingobium chlorophenolicum*, PheA1, phenol hydroxylase from *Bacillus thermoglucosidasius* A7, *Re*TcpA, 2,4,6-trichlorophenol 4-monooxygenase from *Ralstonia eutropha* JMP134, TftD, 2,4,5-trichlorophenol 4-monooxygenase from *Burkholderia cepacia* AC1100, *Tt*HpaB, *p*-hydroxyphenylacetate hydroxylase from *Thermus thermophilus* HB8

## Abstract

HadA is a flavin-dependent monooxygenase catalyzing hydroxylation plus dehalogenation/denitration, which is useful for biodetoxification and biodetection. In this study, the X-ray structure of wild-type HadA (HadA_WT_) co-complexed with reduced FAD (FADH^–^) and 4-nitrophenol (4NP) (HadA_WT_−FADH^–^−4NP) was solved at 2.3-Å resolution, providing the first full package (with flavin and substrate bound) structure of a monooxygenase of this type. Residues Arg101, Gln158, Arg161, Thr193, Asp254, Arg233, and Arg439 constitute a flavin-binding pocket, whereas the 4NP-binding pocket contains the aromatic side chain of Phe206, which provides π-π stacking and also is a part of the hydrophobic pocket formed by Phe155, Phe286, Thr449, and Leu457. Based on site-directed mutagenesis and stopped-flow experiments, Thr193, Asp254, and His290 are important for C4a-hydroperoxyflavin formation with His290, also serving as a catalytic base for hydroxylation. We also identified a novel structural motif of quadruple π-stacking (π-π-π-π) provided by two 4NP and two Phe441 from two subunits. This motif promotes 4NP binding in a nonproductive dead-end complex, which prevents C4a-hydroperoxy-FAD formation when HadA is premixed with aromatic substrates. We also solved the structure of the HadA_Phe441Val_−FADH^–^−4NP complex at 2.3-Å resolution. Although 4NP can still bind to this variant, the quadruple π-stacking motif was disrupted. All HadA_Phe441_ variants lack substrate inhibition behavior, confirming that quadruple π-stacking is a main cause of dead-end complex formation. Moreover, the activities of these HadA_Phe441_ variants were improved by ⁓20%, suggesting that insights gained from the flavin-dependent monooxygenases illustrated here should be useful for future improvement of HadA’s biocatalytic applications.

Halogenated phenols (HPs) and nitrophenol (NP) are persistent pollutants resulting from anthropogenic activities including the use of agro- and household chemicals such as pesticides, herbicides, and flame retardants. Their accumulation in the environment can pose a hazard to human health, with toxic effects ranging from causing chronic diseases to acute death (1). Through natural evolution, microbes have developed enzymes and metabolic pathways to combat against these chemicals by degrading them into common metabolites, which can then be used as cellular energy sources (2, 3). The *had* pathway from *Ralstonia pickettii* is one of the most well-known pathways for biodegrading HPs and NP. It contains several enzymatic reactions catabolizing pesticides such as 2,4-dichlorophenol, 2,4,5-trichlorophenol, and 2,4,6-trichlorophenol to generate benzoquinone (BQ) derivatives, which can be assimilated into the tricarboxylic acid cycle (4, 5, 6, 7, 8).

The initial and committing step of the *had* pathway catalyzed by HadA monooxygenase is hydroxylation with removal of either halide substituents (F, Cl, Br, or I) from HPs or a nitro group (-NO_2_) from NP (6, 7, 8). HadA can detoxify a wide range of toxicants, and its ability to convert monosubstituted HPs and NP to a valuable compound such as D-luciferin has recently been shown (9). The ability of HadA to synthesize D-luciferin highlights its potential value for use in toxicant waste refineries. Rather than allowing HPs or NP to be dissipated into the open environment, thereby creating toxic ecological effects, a proper waste collection and detoxification procedure can be performed using HadA to detoxify and convert them to valuable compounds.

HadA belongs to the class D two-component flavin-dependent monooxygenases, which use reduced FAD (FADH^–^) as a substrate (7, 10, 11, 12, 13, 14). Kinetic mechanisms of wild-type HadA (HadA_WT_) using 4-chlorophenol (4CP), 4-bromophenol, 4-iodophenol, 4-fluorophenol, 4-nitrophenol (4NP), and phenol as substrates have shown that a binary complex of HadA-FADH^–^ is the first species to form before reacting with O_2_ to form a C4a-hydroperoxy-FAD intermediate (Fig. 1) (7, 8). A substrate such as 4CP then binds and receives a terminal –OH group (electrophile) from the C4a-hydroperoxy-FAD, which is incorporated into the C4 position of 4CP (nucleophile) *via* an electrophilic aromatic substitution mechanism, resulting in a C4a-hydroxy-FAD intermediate and the hydroxylated product. Next, a halide or nitro group is eliminated to form benzoquinone as a final product while C4a-hydroxy-FAD is dehydrated to form oxidized FAD before the product is released from HadA to complete the catalytic cycle. The quantitative structure–activity relationship analysis revealed that the overall reaction of HadA is controlled by the ability of the substrate to be deprotonated because the rate constants of HadA reactions with substrates having different substituents at the 4-position depend directly on the p*K*_a_ values of the compounds (8).

**Figure 1:**
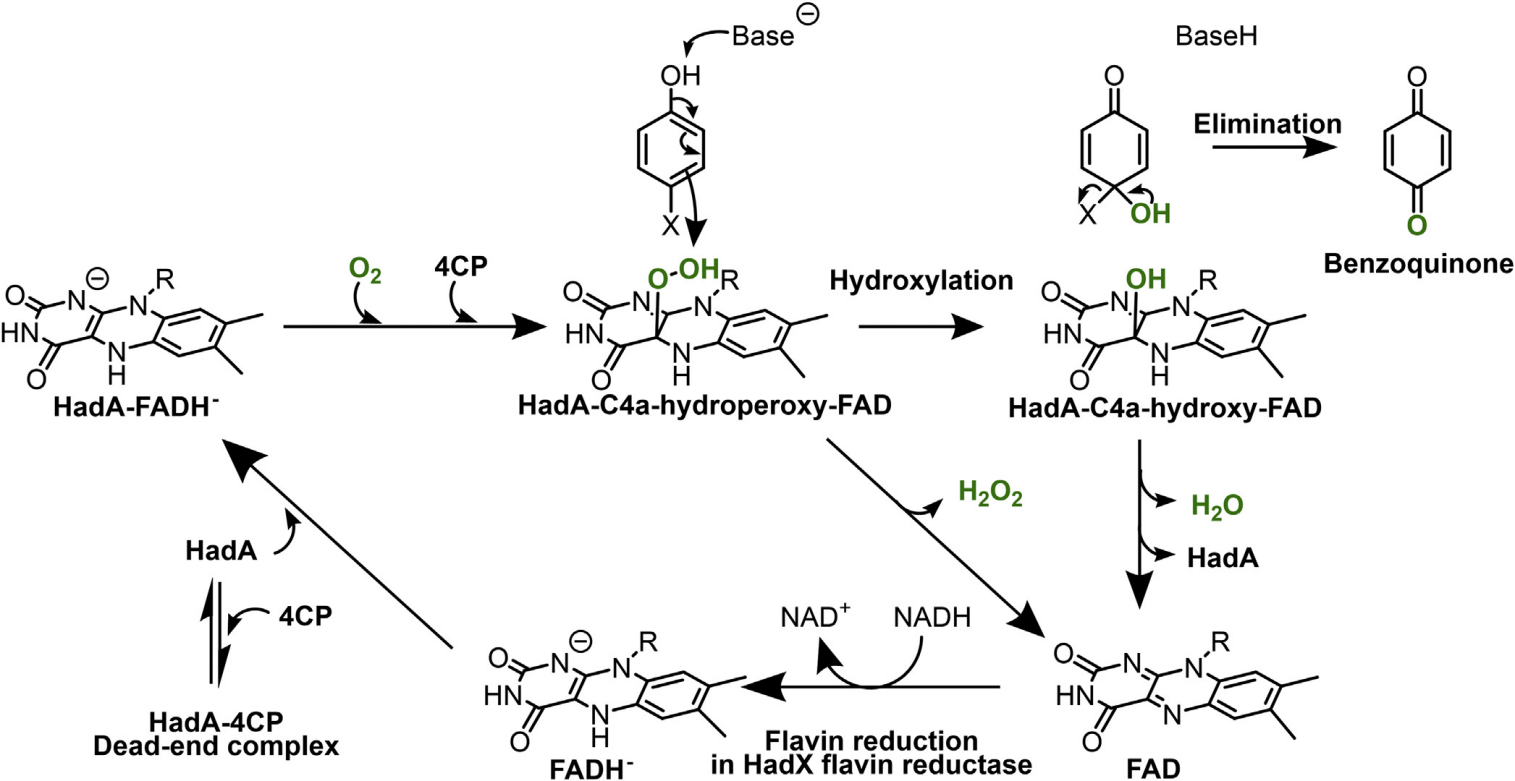
**The overall reaction mechanism of HadA monooxygenase** (6, 7, 8).

Despite its potential use in biodetoxification and known reaction mechanisms, the X-ray crystallographic structures of HadA or related homologs in complex with flavin and aromatic substrates have not been reported. Only structures of apo-HadA_WT_ and its homologs including apo-*Re*TcpA from *Ralstonia eutropha* JMP134 and apo-TftD from *Burkhodelria cepacia* AC1100 without bound ligand were reported (15, 16, 17). Thus, insights into the substrate recognition and catalytic mechanism of HadA are still not understood.

The reaction of HadA also has a side pathway that impedes the application of this enzyme in real and scalable usage. HadA_WT_ displays strong substrate inhibition by the preformed HadA_WT_–4CP complex or “dead-end complex,” which prevents formation of C4a-hydroperoxy-FAD and thus inactivates the overall reaction (Fig. 1) (7). We note that substrate inhibition by formation of a dead-end complex is also found in other two-component flavin-dependent monooxygenases such as bacterial luciferase (class C) (18, 19, 20). However, no structural features that can promote substrate inhibition in these enzymes are known. This phenomenon limits the enzyme usage in biocatalysis because the flavin and substrate need to bind to the enzyme in a strict sequential order to avoid inhibition. This can be achieved under low substrate concentrations because the rate of flavin binding is generally faster. However, under high substrate concentrations, the rate of substrate binding (bimolecular reaction), which is dependent on the concentration, can be faster than the flavin binding. This can cause the turnovers to cease (7). Therefore, molecular insights into the mode of substrate inhibition gained by structural analysis of HadA would be useful for shedding light on the mechanisms underlying catalysis and inhibition and would allow improvement in applications of HadA and other two-component flavin-dependent monooxygenases in general.

In this work, we present the crystal structures of a ternary complex of HadA_WT_ and FADH^–^ and 4-nitrophenol (HadA_WT_–FADH^–^–4NP), representing the first comprehensive ligand-bound structure of dehalogenating/denitrating flavin-dependent monooxygenases. The binding pocket of substrates 4NP and FADH^–^ was identified, and the interactions of the substrates with the surrounding and key catalytic residues that enable HadA catalysis were determined by site-directed mutagenesis, stopped-flow experiments, and product analysis. Moreover, we found rigid quadruple π-stacking (π-π-π-π) interactions between the aromatic moieties of the two bound 4NP molecules and the two aromatic side chains of Phe441 from two subunits at the dimer interface. This unusual π-π-π-π interaction explains the root cause of substrate inhibition by forming a dead-end complex. We verified this hypothesis by disrupting the quadruple π-stacking interaction by site-directed mutagenesis of Phe441. The results indeed showed that the substrate inhibition was alleviated in the HadA_Phe441Val_ and HadA_Phe441Leu_ variants. This is the first time that the substrate inhibition mechanism in flavin-dependent monooxygenases can be explained structurally. The outcome of this work should help improve future applications of flavin-dependent monooxygenases in general.

## Results

### The overall structure of the HadA_WT_−FADH^–^−4NP ternary complex

**Figure 2:**
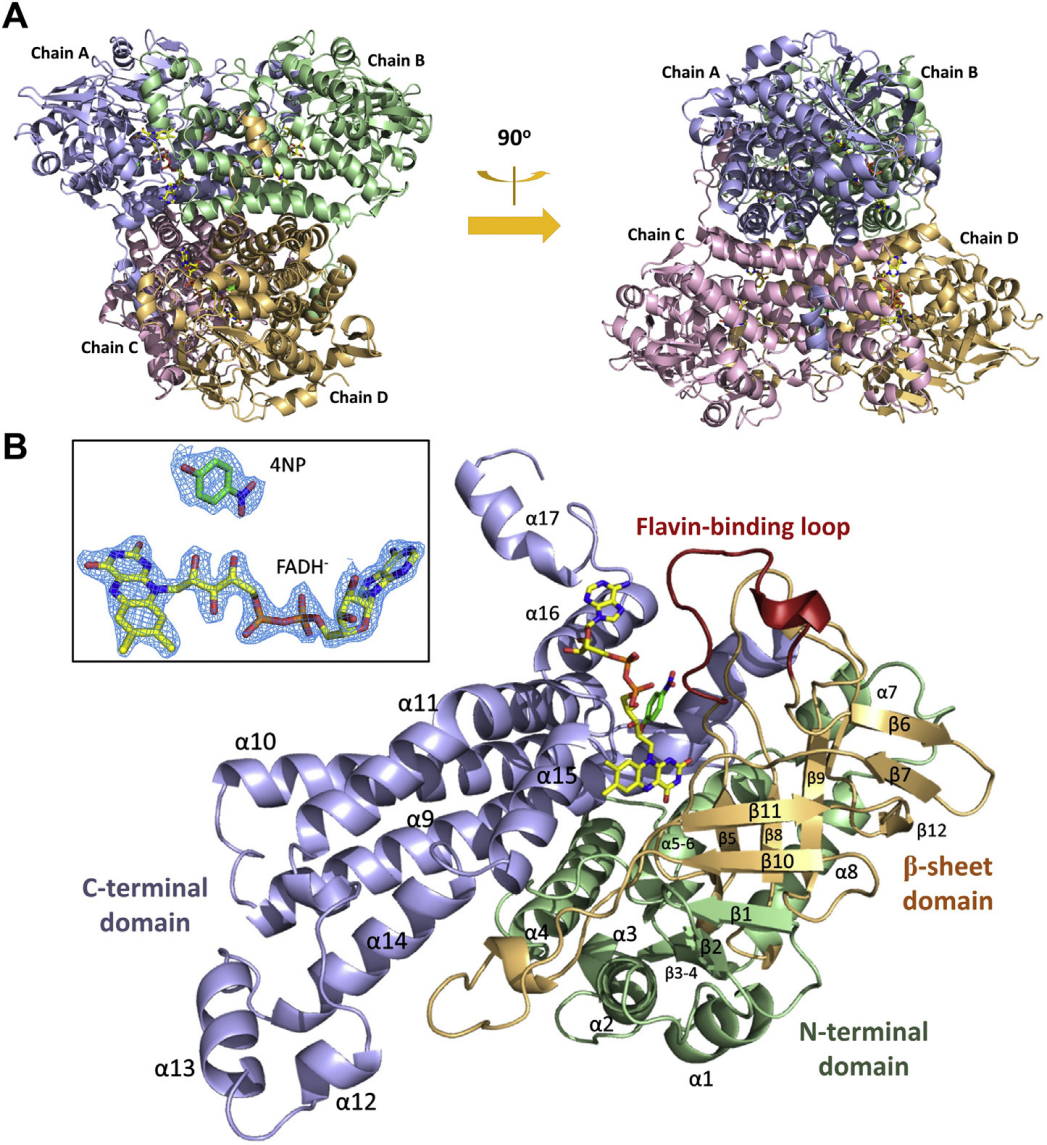
**Crystal structure of the HadA**_**WT**_**–FADH**^**–**^**–4NP complex.***A*, tetrameric quaternary structure of HadA monooxygenase co-complexed with FADH^–^ and 4NP. *B*, three domains of the HadA subunit include the N-terminal domain (*green part*), the β-sheet domain (*orange part*), and C-terminal domain (*purple part*). The flavin-binding loop (residues 157–170) is labeled in red. *Inset* in (*B*) is an electron density map of FADH^–^ and 4NP bound in the active site. 4NP, 4-nitrophenol.

In order to gain structural and mechanistic insights into HadA, we cocrystallized the enzyme with FADH^–^ and 4NP to obtain the first ternary structure of HadA_WT_−FADH^–^−4NP at a 2.3-Å resolution. Data and refinement statistics are listed in Table 1. The HadA_WT_−FADH^–^−4NP ternary complex (Protein Data Bank [PDB] code: 7E8P) adopts a common structural fold of the acyl-CoA dehydrogenase flavoenzyme family with two dimers associated to form a tetramer (Fig. 2*A*), similar to that of apo-HadA_WT_ (PDB code: 6JHM) (RMSD of 0.439) previously reported (Fig. S1) (16). Each protomer is composed of three domains: the N-terminal domain (residues 1–146), the β-sheet domain (residues 147–275), and the C-terminal domain (residues 276–517) (Fig. 2*B*). The co-complex with FADH^–^ and 4NP allowed identification of four binding sites for ligands in which strong electron densities could be observed for all four FADH^–^ molecules, whereas only one 4NP molecule could be clearly seen in the expected pocket of subunit A (Fig. 2*B*, *Inset*). Of interest, two 4NP molecules were found to locate to the dimer interface between subunits C and D. The key difference between the HadA_WT_−FADH^–^−4NP structure reported here and the previously reported apo-HadA_WT_ structure is the loop of residues 157 to 170, which assumes a distinct structure in the ternary complex, whereas it is disordered in the apo-HadA_WT_. In contrast, the C-terminal helix α19, which can be clearly observed in the apo-HadA_WT_, is disordered in the ternary complex (Figs. S1 and S2).

**Table 1:** Summary of crystallographic data of HadA complexes

Complex	HadA_WT_–FADH^–^–4NP	HadA_Phe441Val_–FADH^–^–4NP
Wavelength (Å)	1.54	1.54
Space group	*P*2_1_2_1_2_1_	*P*2_1_2_1_2_1_
Unit cell parameters *a,b,c* (Å) *α,β,γ* (^o^)	*a* = 97.8, *b* = 161.9, *c* = 168.6, *α,β,γ* = 90	*a* = 97.8, *b* = 161.6, *c* = 168.6, *α,β,γ* = 90
Molecules in asymmetric unit	4	4
Resolution (Å)	20.74–2.30 (2.40–2.30)	21.10–2.30 (2.40–2.30)
No. of measured reflections	586,953	635,201
No. of unique reflections	118,305	118,765
Redundancy	4.9 (3.4)	5.3 (4.0)
Completeness (%)	99.1 (98.4)	99.6 (98.8)
<*I/σ(I)*>	10.2 (2.8)	19.4 (6.7)
R_merge_ (%)	10.7 (34.2)	6.2 (16.1)
Wilson B factor (Å^2^)	17.5	13.7
R_factor_/R_free_ (%)	20.71/26.25	16.63/20.99
Average *B*-factor (Å^2^)	20.9	15.1
R.m.s. bond deviation (Å)	0.01	0.01
R.m.s. angle deviation (^o^)	1.67	1.65
Favored, Allowed, Outliers	90.6, 9.1, 0.2	91.4, 8.4, 0.2
PDB code	7E8P	7E8Q

### The overall FADH^–^-binding site of HadA_WT_

**Figure 3:**
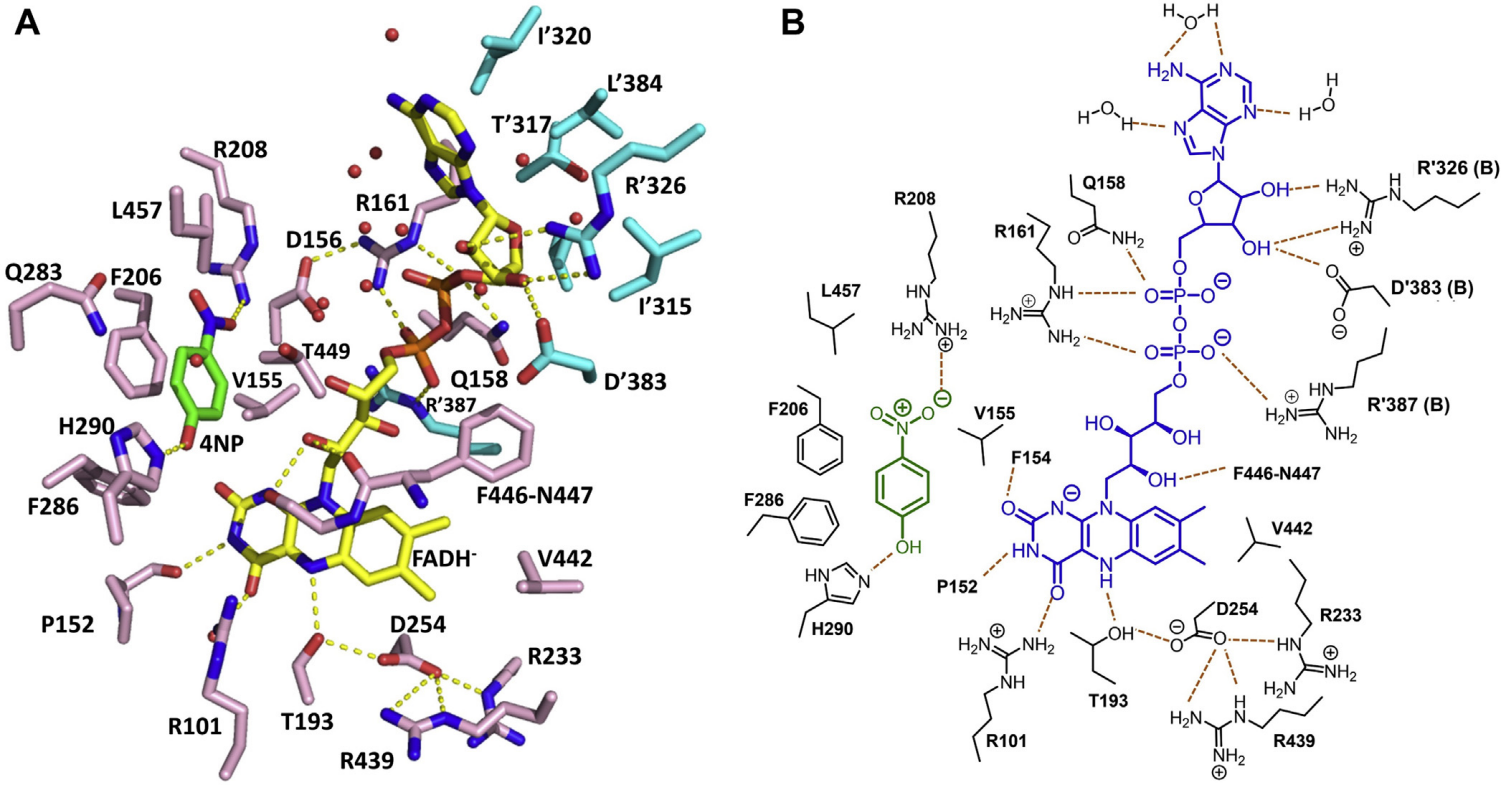
**Binding interactions of FADH**^**–**^**and 4NP within the active site of HadA**_**WT**_**.***A*, amino acid residues and interactions at the binding pockets of 4NP, and adenosine and isoalloxazine moieties of FADH^–^. FADH^–^ is shown in *yellow*, whereas 4NP is shown in *green*. *Pink* residues indicate amino acids from subunit A, whereas *cyan* residues indicate amino acids from subunit B. *B*, a simple scheme to illustrate interactions of 4NP and FADH^–^ with residues in the active site of HadA_WT_. 4NP, 4-nitrophenol.

FADH^–^ binds to each subunit in a tabular-shaped pocket, created by the N-terminal and β-sheet domains, and extends to the C-terminal domain. The adenosine moiety protrudes out toward the dimer interface and is stabilized by polar interactions and hydrophobic residues of the dimer (Fig. 3*A*). The loop of residues 157 to 170 (Fig. 2*B*) forms a lid to confine the bound FADH^–^ *via* interactions with an adenosine diphosphate moiety. This loop is designated as the “flavin-binding loop” that was previously identified in other two-component flavin-dependent enzymes (21, 22). Gln158 and Arg161, which are parts of the flavin-binding loop, and Arg′387 (from a neighboring subunit) anchor the diphosphate backbone *via* salt-bridge and hydrogen-bonding interactions. The geometry of these arginine side chains is strengthened by salt-bridge interactions with Asp156. Similar to the structure of *p*-hydroxyphenylacetate (4HPA) hydroxylase from *Thermus thermophilus* HB8 (*Tt*HpaB-FAD-4HPA), the diphosphate moiety of the ribityl side chain of FAD is hydrogen bonded to side chains of the conserved glutamine and arginine, stabilizing the loop closure upon FADH^–^ binding (22). In contrast, this loop was not observed in the structure of *p*-hydroxyphenylacetate hydroxylase from *Acinetobacter baumannii* (C_2_–FMNH^–^–4HPA) (23), in which C_2_ can bind well to all types of flavins including FMNH^–^, FADH^–^, and reduced riboflavin (24, 25, 26). Therefore, this structural feature may be a universal motif specific for selectively binding FADH^–^ in two-component flavin-dependent monooxygenases.

The ribose moiety of adenosine is hydrogen bonded to Arg′326 and Asp′383 in the C-terminal domain of the neighboring subunit of the dimer, whereas the adenine ring resides within the van der Waals contacts of Ile′315, Thr′317, Ile′320, Val′323, Leu′384 of the neighboring C-terminal domain and in a water tunnel of the tetrameric inner-interface. The 2D scheme for HadA–FADH^–^ interactions in the presence of 4NP within its active site is shown in Figure 3*B*.

### Interactions around the flavin isoalloxazine ring in the structure of HadA_WT_−FADH^–^−4NP

The isoalloxazine ring of FADH^–^ binds deeply at the base of the tabular pocket and interacts with residues of both subunits of the dimer (Fig. 3*A*). The aromatic ring of the isoalloxazine moiety makes van der Waals contacts with Ile191, Val442, Phe443, Phe446, and Arg′387 and Ile′391 of the neighboring subunit, whereas the ribityl side chain forms hydrogen bonds to the backbones of Phe446 and Asn447. The 2-carbonyl (O2) and 3-NH(H(N3)) groups of the isoalloxazine are hydrogen bonded to the amino and carboxyl backbones of Phe154 and Pro152 with distances of 2.8 and 3.1 Å, respectively, whereas the 4-carbonyl (O4) and 5-NH(H(N5)) are fixed by hydrogen bonding from side chains of Arg101 (2.7 Å) and Thr193 (3.1 Å), respectively. The position of a guanidine side chain of Arg101 is controlled by a nearby Asn447 side chain (3.0 Å). The hydroxyl group of Thr193 is 2.7 Å from the carboxyl ion of Asp254, which is salt bridged to the guanidine side chains of Arg233 and Arg439. This FADH^–^ configuration suggests that a molecular O_2_ would diffuse to reach the C4a atom on the *re*-face to form C4a-hydroperoxy-FAD; this geometric mode of oxygen reaction is also commonly found in other two-component flavin-dependent monooxygenases (27).

The interactions contributed by Thr193 together with Asp254, Arg233, and Arg439 should be important for the flavin chemistry of HadA, as they are directly involved in the alteration of the hybridization of the isoallozaxine N5 during the reaction. To identify the functions of these residues, four HadA_Thr193_ and two HadA_Asp254_ variants were constructed by site-directed mutagenesis (listed in Table. S1). Three HadA_Thr193_ variants, including HadA_Thr193Ala_, HadA_Thr193Val_, and HadA_Thr193Ser_, could be expressed in soluble form. In contrast, HadA_Thr193Cys_ could only be expressed in inclusion bodies. Both HadA_Asp254_ variants of Asp254Ala and Asp254Asn could also be expressed in soluble form. Thus, the catalytic properties of the soluble enzymes were further characterized by monitoring 4NP consumption and by performing transient kinetics studies (Experimental procedures).

**Figure 4:**
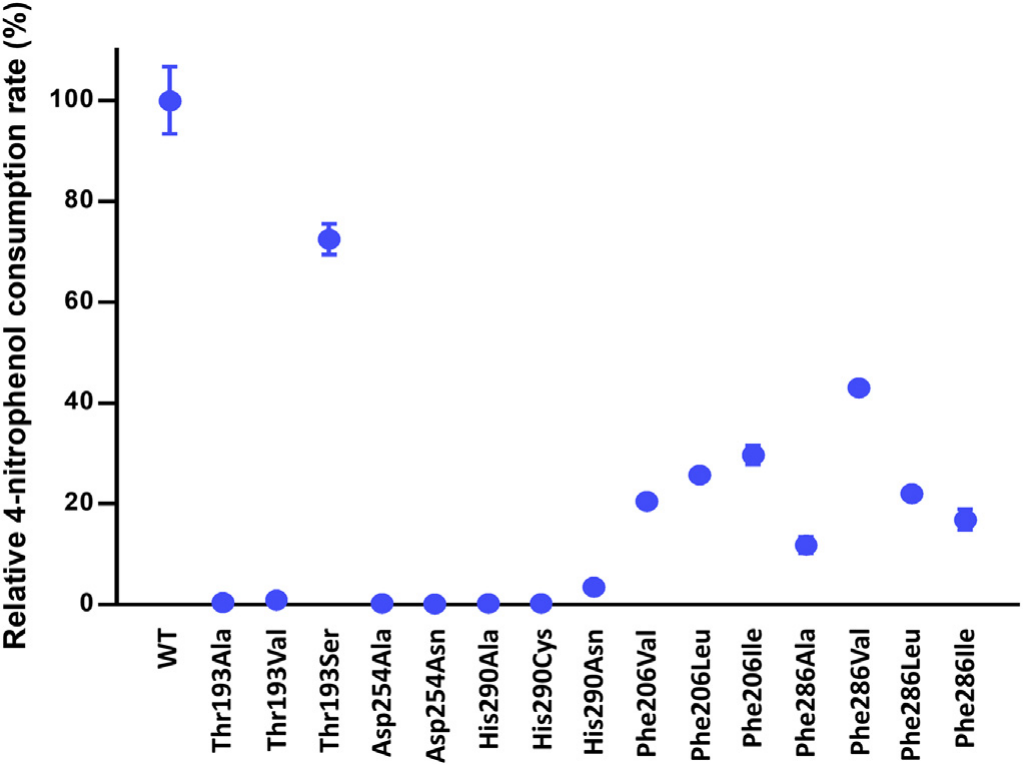
**Relative 4NP consumption activities of HadA variants.** Multiple turnover reactions consisting of 4-nitrophenol or 4NP (50 μM), Glc-6-P (1 mM), Glc-6-PD (0.5 unit/ml), NAD^+^ (5 μM), HadX (1 μM), HadA variants (10 μM) in 20 mM Hepes pH 7.5 were carried out. UV-visible spectra were monitored to observe the decrease of absorption around the 400-nm region due to loss of the 4NP substrate. 4NP consumption activity of HadA_WT_ was set to 100% for comparison with Thr193, Asp254, His290, Phe206, and Phe286 variants.

**Figure 5:**
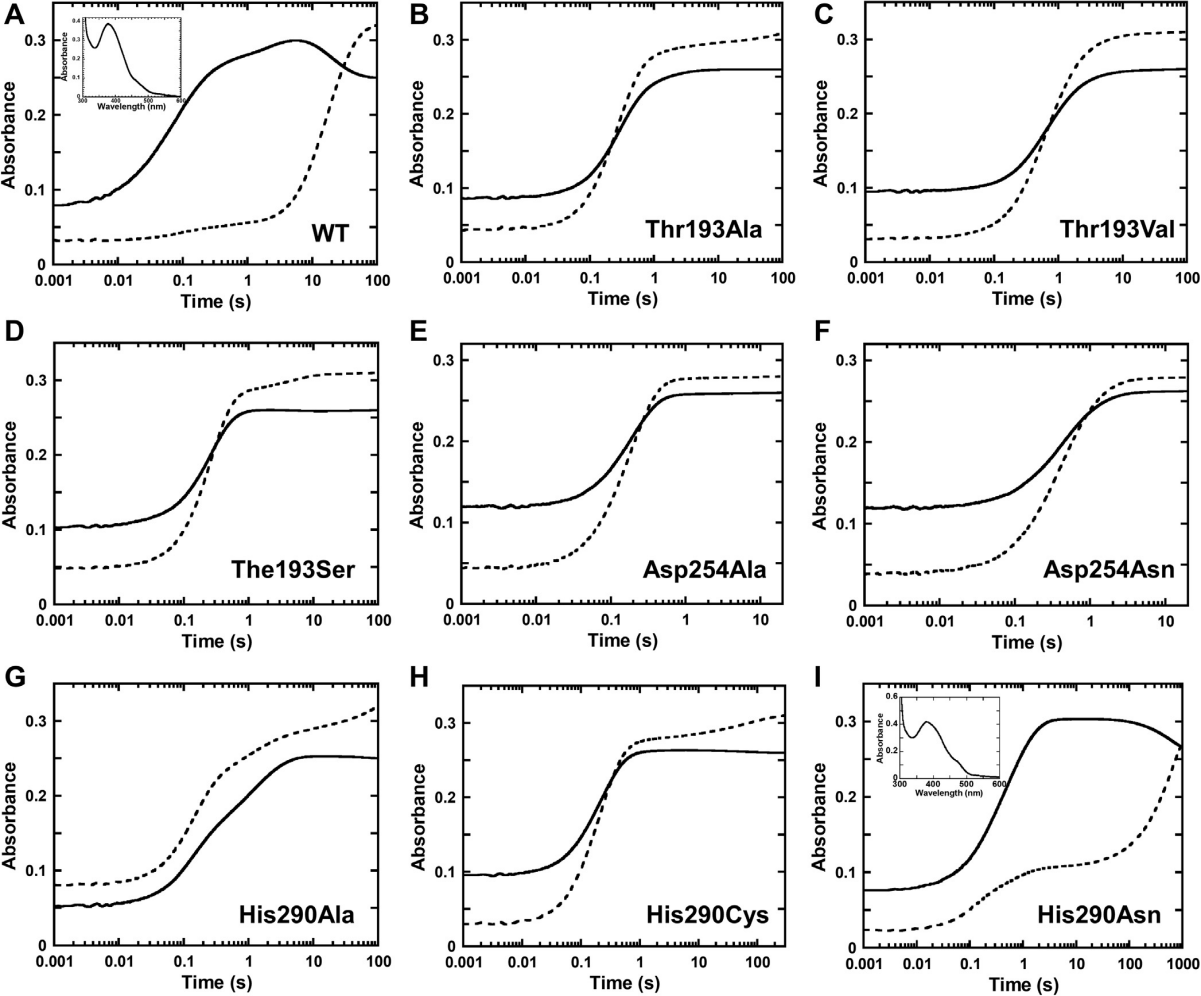
**Transient kinetics of HadA variants.** Rapid kinetics of reactions of HadA_variant_–FADH^–^ (75 and 25 μM, respectively) mixing with an aerobic solution of 4-chlorophenol (0.5 mM 4-chlorophenol and 0.13 mM O_2_) using the single mixing mode of a stopped-flow apparatus. Absorption changes at wavelengths 380 nm (solid line) and 450 nm (dashed line) were monitored to observe the C4a-hydroperoxy-FAD intermediate and oxidized FAD, respectively. C4a-hydroperoxy-FAD absorbs mainly at *A*_380_, whereas oxidized FAD absorbs at both wavelengths. HadA variants are (*A*) HadA_WT_, (*B*) HadA_Thr193Ala_, (*C*) HadA_Thr193Val_, (*D*) HadA_Thr193Ser_, (*E*) HadA_Asp254Ala_, (*F*) HadA_Asp254Asn_, (*G*) HadA_His290Ala_, (*H*) HadA_His290Cys_, and (*I*) HadA_His290Asn_. *Inset* in (*A*) and (*I*) are spectra of the C4a-hydroperoxy-FAD intermediate detected at 10 s of reaction time.

Results showed that HadA_Thr193Ala_ and HadA_Thr193Val_ could not utilize 4NP as a substrate (Fig. 4). Kinetic data of HadA_Thr193Ala_ and HadA_Thr193Val_ revealed that only free FADH^–^ oxidation (Fig. S3) was observed when mixing FADH^–^ with these enzymes, indicating impairment in their FADH^–^ binding. These two variants did not show C4a-hydroperoxy-FAD formation as in the HadA_WT_ (Fig. 5, *A*–*C*). For the HadA_Thr193Ser_ variant, its 4NP consumption activity was ⁓72% relative to HadA_WT_ (Fig. 4). Kinetic traces of the HadA_Thr193Ser_ reaction with aerobic 4CP (Fig. 5*D*) revealed two observable phases (0.01 – 1s and 1s – 100 s) when detected at wavelength 450 nm (*A*_450_). These kinetic phases indicate oxidations of free FADH^–^ (84%) and the HadA-bound FADH^–^ (16%) species. A small amount of C4a-adduct formation could be observed at wavelength 380 nm (*A*_380_). Therefore, the HadA_Thr193Ser_ variant could maintain partial ability to bind FADH^–^ but with less efficiency than HadA_WT_. This indicates the importance of the hydroxyl group of residue 193, which is required for interacting with H(N5) of FADH^–^. Therefore, we propose that the -OH group of Thr193 directly interacts with H(N5) of FADH^–^ for binding and stabilization of FADH^–^ and for formation of its intermediates.

Apart from Thr193, Asp254 is also important for FADH^–^ binding. Disruption of hydrogen bonding networks in the variants HadA_Asp254Ala_ and HadA_Asp254Asn_ also resulted in complete loss of 4NP consumption activity (Fig. 4). Based on kinetic data, these variants also lose FADH^–^ binding ability because only free FADH^–^ oxidation was observed in their reactions (Fig. 5, *E* and *F*). We propose that hydrogen bonding and salt-bridge interactions between the Thr193 side chain and neighboring residues such as Asp254, Arg233, and Arg439 may render greater negative charge on the hydroxyl group of Thr193, leading to stronger interactions with H(N5) on the *si*-face of the isoalloxazine ring.

### A unifying FADH^–^ binding feature found in HadA and other two-component flavin-dependent monooxygenases

To analyze whether the nature of the amino acids found in the FADH^–^-binding pocket of the ternary complex structure of HadA is also common in other group D monooxygenases, amino acid sequences and structures of these enzymes were analyzed. Sequences of (1) flavin-dependent monooxygenases catalyzing hydroxylation plus dehalogenation and denitration activities (HadA monooxygenase from *R. pickettii* [this study]); (2) flavin-dependent monooxygenases catalyzing hydroxylation plus dehalogenation (*Re*TcpA from *R. eutropha* JMP134 (15, 28), *Cn*TcpA from *Cupriavidus nantongensis* X1^T^ (29), TftD from *B. cepacia* AC1100 (17, 30), CphC-I from *Arthrobacter chlorophenolicus* A6 (31), DcmB1 from *Rhodococcus* sp. JT-3 (32), and HnpA from *Cupriavidus* sp. CNP-8 (33)); (3) flavin-dependent monooxygenases catalyzing hydroxylation plus denitration (NpcA from *Rhodococcus opacus* SAO101 (34), NpdA2 from *Arthrobacter* sp. Strain JS443 (35), and NpsA1 from *Rhodococcus* sp. strain PN1 (36)); and (4) flavin-dependent monooxygenases catalyzing merely hydroxylation of phenolic compounds such as phenol and *p*-hydroxyphenylacetate (*Tt*HpaB from *T. thermophilus* HB8 (22), *Ec*HpaB from *Escherichia coli* (37), PheA1 from *Bacillus thermoglucosidasius* A7 (38), and C_2_ from *A. baumannii* (24, 26)) were analyzed by the Clustal omega software (EMBL-EBI) (Fig. S4). Sequences that are similar to, or different from, HadA were identified. Their available enzyme X-ray structures were also compared with that of HadA to identify structural features associated with specific functions. Particularly, the C_2_ structure is important for identifying features specific for FADH^–^ binding because unlike other enzymes, C_2_ can use FADH^–^ and FMNH^–^ equally well (24, 25, 26).

For residues responsible for FADH^–^ recognition, the analysis revealed that the residues Gln158 and Arg161 located in the “flavin-binding loop” (Figs. 2*B* and 3*A*), which interact directly with the diphosphate backbone of the adenosine diphosphate ribityl side chain, are conserved in all two-component FADH^–^-utilizing enzymes, but not in C_2_, which can also bind to FMNH^–^. Therefore, we propose that these residues are important for discriminating between FADH^–^ and FMNH^–^ binding and that they are an important feature responsible for the enzyme selectivity for only binding FADH^–^.

**Figure 6:**
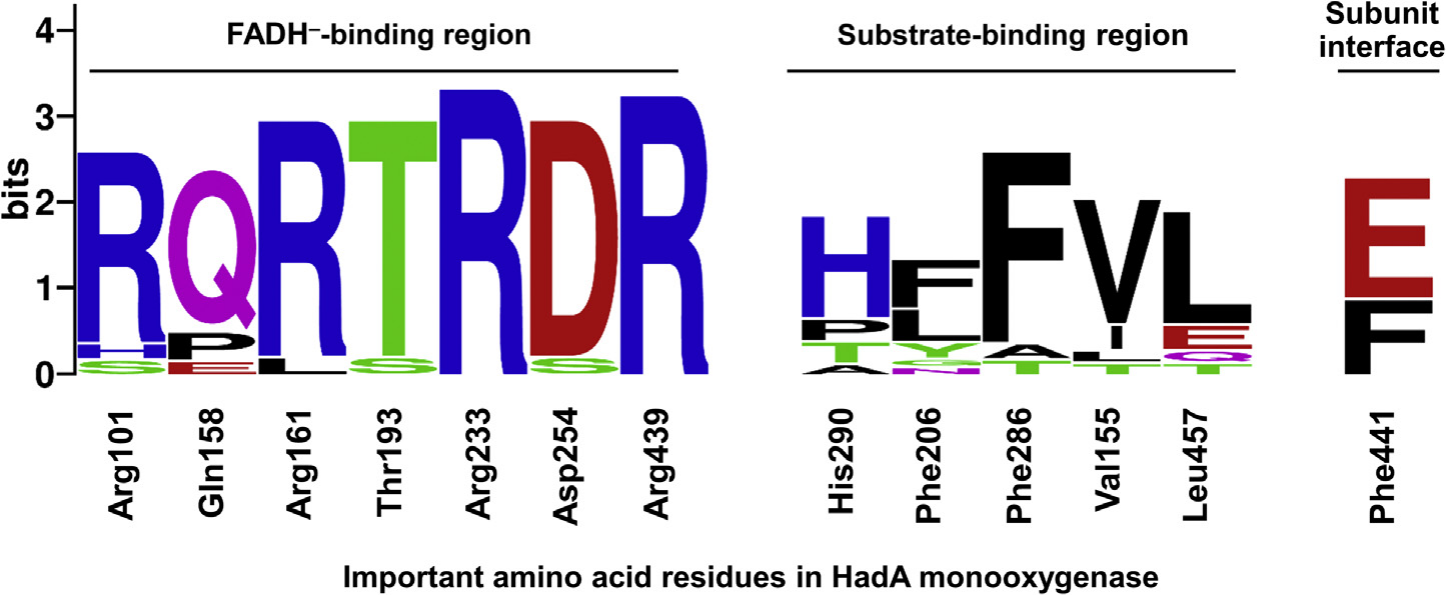
**Conserved amino acid residues of group D two-component monooxygenases.** Important residues in HadA_WT_ including the FADH^–^-binding region, substrate-binding region, and subunit interface were analyzed by Clustal omega (EMBL-EBI). The figure was generated by WebLogo.

For residues surrounding the isoalloxazine ring, all FADH^–^-utilizing monooxygenases in class D have residues equivalent to Arg101, Thr193, Arg233, Asp254, and Arg439 of HadA and their structural configurations are also conserved in the same manner (15, 16, 17, 21, 22) (Fig. S5, *A–E*). This is different from C_2_ in which Ser171 (in C_2_) instead of threonine was identified as being important for FMNH^–^ binding and C4a-hydroperoxy intermediate stabilization by H-bond interactions with both H(N5) and O4 of FMNH^–^ (39). The hydroxyl side chain of Ser171 is stabilized by hydrogen bonding with two adjacent serine residues (23) instead of the salt bridge found in HadA (Fig. S5*F*). Although reactions of HadA homologs have not been investigated by transient kinetics, we can infer that these conserved residues likely function similarly to those of HadA in that they are important for facilitating the reaction of FADH^–^ and oxygen. Residues of HadA important for its catalytic functions are highlighted in the figure generated by WebLogo (40) (Fig. 6).

### Identification of hydrophobic interactions for 4NP binding

In the active site of HadA, the 4NP molecule binds in a hydrophobic pocket (consisting of Val155, Phe206, Phe286, Thr449, and Leu457) located between the β-sheet and C-terminal domains of each subunit. The aromatic ring of 4NP forms contacts with Phe206 face-to-face with π-π stacking and sits in van der Waals contact of Phe286, which forms π-edge interactions with Phe206 (Fig. 3). The hydrophobic environment found in the HadA active site is similar to the environment of an empty pocket speculated to be the phenol-binding pocket in apoenzyme structures of dehalogenating flavin-dependent enzymes previously crystallized (Fig. S6) (15, 16, 17).

We tested the importance of hydrophobic residues surrounding the 4NP-binding pocket using site-directed mutagenesis to decrease the hydrophobicity of the pocket *via* replacing the aromatic side chain of Phe206 and Phe286 with aliphatic side chains of Val, Leu, Ile, or Ala. The variants HadA_Phe206Val_, HadA_Phe206Leu_, HadA_Phe206Ile_, HadA_Phe286Ala_, HadA_Phe286Val_, HadA_Phe286Leu_, and HadA_Phe286Ile_ showed a drastic decrease in 4NP conversion activities (Fig. 4). Molecular docking of 4NP into the active sites of HadA_WT_ and Phe206 and Phe286 variants of HadA performed by AutoDock Vina to determine the binding energy (Experimental Procedures) showed that the affinities of 4NP in HadA_Phe206_ variants decreased because the π-π stacking interactions between 4NP and Phe206 were lost. Although the binding affinities of 4NP in the HadA_Phe286_ variants were not significantly perturbed (Fig. S7), the denitration activities of these HadA_Phe286_ variants were impaired. The data suggest that, in addition to the ability to bind, proper geometric arrangement of 4NP in the active site is also important for hydroxylation and denitration of 4NP by HadA. Altogether, we demonstrated that the aromatic side chains of Phe206 and Phe286 are important for promoting the correct configuration of 4NP binding in HadA catalysis.

When apoenzyme structures of enzymes (*Re*TcpA and TftD) with similar activities to HadA were analyzed, the residues Phe206 and Phe286, along with other hydrophobic residues such as Val155 and Leu457, could be identified. Based on this information and the HadA structure, we propose the substrate-binding regions of these enzymes as shown in Figures 6 and S4.

### His290 is a key catalytic residue for electrophilic aromatic substitution

In the 4NP-binding site, a polar hydroxyl group of 4NP interacts closely with His290 (2.5 Å) on helix α9, whereas the nitro (-NO_2_) moiety of 4NP points toward a guanidine side chain of Arg208 (3.4 Å). Based on apoenzyme structures, His290 in HadA and His289 in TftD were previously proposed as a general base to abstract a proton from a phenolic substrate, which triggers delocalization of a lone pair of electrons from O1 to the C4 position of the substrate, facilitating monooxygenation by C4a-hydroperoxy-FAD (shown in Fig. 1) (8, 17, 41). To elucidate the role of His290 in catalysis, we constructed and overexpressed nine His290 variants (listed in Table. S1). Only HadA_His290Ala_, HadA_His290Cys_, and HadA_His290Asn_ could be overexpressed as soluble enzymes, and these enzymes were purified to homogeneity. HadA_His290Ala_ and HadA_His290Cys_ exhibited a complete loss of 4NP conversion activity, whereas HadA_His290Asn_ showed a very low 4NP consumption activity (Fig. 4).

We carried out stopped-flow investigation of these three variants and found that transient kinetics of HadA_His290Ala_ and HadA_His290Cys_ showed mixed kinetics of free and enzyme-bound FADH^−^ oxidation (Fig. 5, *G* and *H*). However, formation of C4a-hydroperoxy-FAD could not be detected in these variants, explaining why these enzymes are completely inactive. Of interest, the kinetic traces of the HadA_His290Asn_ reaction showed three observable phases (Fig. 5*I*). The first phase (0.001–0.4 s) showed simultaneous increase in *A*_380_ and *A*_450_ with an observed rate constant of ∼6 s^–1^ similar to kinetics of free FADH^–^ oxidation (Fig. S3). This phase was interpreted as oxidation of unbound FADH^–^. The second phase (0.4–20 s) exhibited only a large increase in *A*_380_ with almost no change in *A*_450_. This phase likely corresponded to formation of a C4a-hydroperoxy-FAD intermediate with an observed rate constant of ∼2 s^–1^. The absorption characteristics of the intermediate formed at this phase fits well with that of C4a-hydroperoxy-FAD (Fig. 5*I*, *inset*). The last phase was an increase of *A*_450_ and decrease of *A*_380_, indicating that this phase was likely a decay of the C4a-hydroperoxy-FAD intermediate with generation of oxidized FAD with an observed rate constant of ∼0.002 s^–1^. The decay rate of C4a-hydroperoxy-FAD in HadA_His290Asn_ (0.002 s^–1^) is in the same range as that of C4a-hydroperoxy-FAD decay in HadA_WT_ (0.007 s^–1^) (7). We could not detect product from a single turnover reaction of HadA_His290Asn_:FADH^–^ carried out in an aerobic solution of 4CP (Experimental Procedures) (data not shown). This suggests that, although C4a-hydroperoxy-FAD can be formed in HadA_His290Asn_, the enzyme mostly eliminates H_2_O_2_ *via* an unproductive pathway without catalyzing dehalogenation/denitration, possibly due to the lack of His290. Therefore, we conclude that His290 is important for stabilization of C4a-hydroperoxy-FAD as well as for facilitating product formation in dehalogenation/denitration reactions as previously proposed (8, 17, 41).

Of interest, His290 is only conserved among monooxygenases with additional dehalogenation and denitration activities but not in monooxygenases catalyzing only hydroxylation (Fig. 6 and Fig. S4). Therefore, this histidine residue should be key for enabling dehalogenation and denitration reactions. Its position is also different from the catalytic base found in enzymes catalyzing *ortho*-hydroxylation of 4HPA, such as Tyr104 and His142 for *Tt*HpaB (22) and His120 for C_2_ (42).

### Proposed mechanisms of hydroxylation and group elimination by HadA

**Figure 7:**
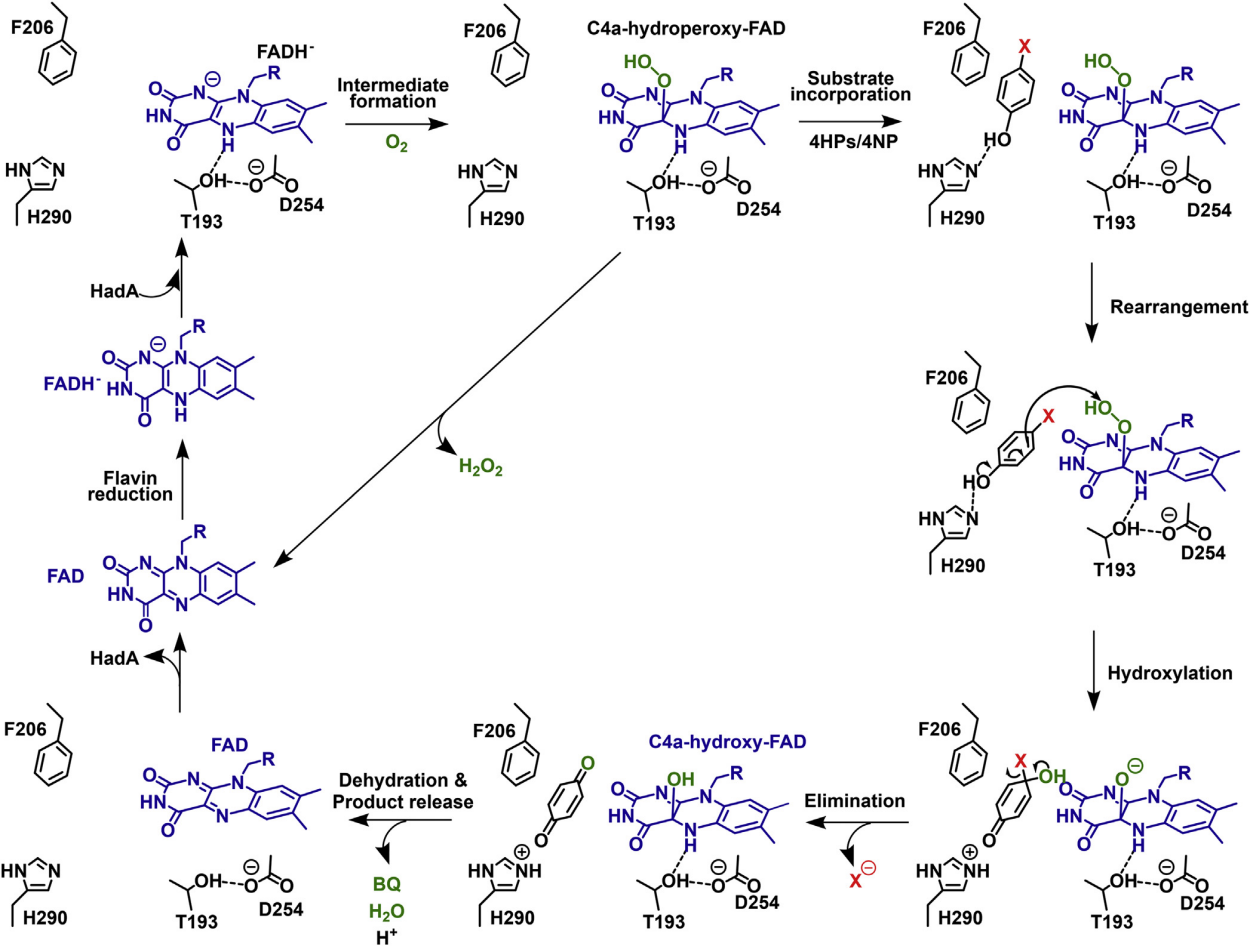
**Proposed reaction mechanisms of HadA monooxygenase with halogenated phenols (HPs) or nitrophenol (NP) bound in its active site**.

The combined results from structural analysis and site-directed mutagenesis experiments suggest a possible model for HadA reaction mechanisms as described in Figure 7. In the first step, FADH^–^ binds to HadA such that the flavin H(N5) forms a hydrogen bond to the Thr193 hydroxyl side chain in which its hydroxy dipole is polarized by the hydrogen bond network of the neighboring residues Asp254, Arg233, and Arg439. The next step is diffusion of O_2_ toward the *re*-side of FADH^–^ to form a C4a-hydroperoxy-FAD intermediate. The substrate then binds to the aromatic cage formed by Phe206 and Phe286 with its C4 position (*para*-position) pointing toward the flavin C4a position.

We noted that, in the HadA_WT_ complex structure (Fig. 3), the substrate C4 position is 9 Å away from the C4a position of FADH^–^, which is significantly farther than the distance between the substrate hydroxylation site and the C4a-position in C_2_ and *Tt*HpaB structures (5.0 and 4.9 Å, respectively) (22, 23). We propose that significant conformational changes in the HadA structure possibly occur after C4a-hydroperoxy-FAD is formed to allow the terminal –OOH moiety of C4a-hydroperoxy-FAD to move closer to the C4 position of the substrate. Based on our previous kinetic investigation (7, 8), we found that, for active hydroxylation, the substrate should bind after C4a-hydroperoxy-FAD is formed. Therefore, the structure obtained likely does not represent the near attack conformation. Subsequently, the flavin, 4NP, and residues around the active site need to be rearranged in order to accommodate an oxygen molecule used for the formation of C4a-hydroperoxy-FAD. Furthermore, the 4NP substrate position needs to be reoriented in order to be ready for reacting with the C4a-hydroperoxy FAD intermediate.

For the hydroxylation step, His290 abstracts a proton from the hydroxyl group of a phenolic substrate to facilitate hydroxylation and group elimination to form BQ and C4a-hydroxy-FAD as products. Our previous investigation on the mechanism of HadA_WT_ has shown that the deprotonation of aromatic substrates is important for controlling HadA catalysis (8). As His290 is the only residue surrounding 4NP that has the potential to act as a catalytic base at the working pH (pH 7.5), it is the most probable candidate responsible for substrate deprotonation. Dehydration of C4a-hydroxy-FAD occurs prior to release of both BQ and oxidized FAD from the HadA active site to complete the catalytic cycle.

### Unusual 4NP-binding site at the subunit interface of HadA_WT_

**Figure 8:**
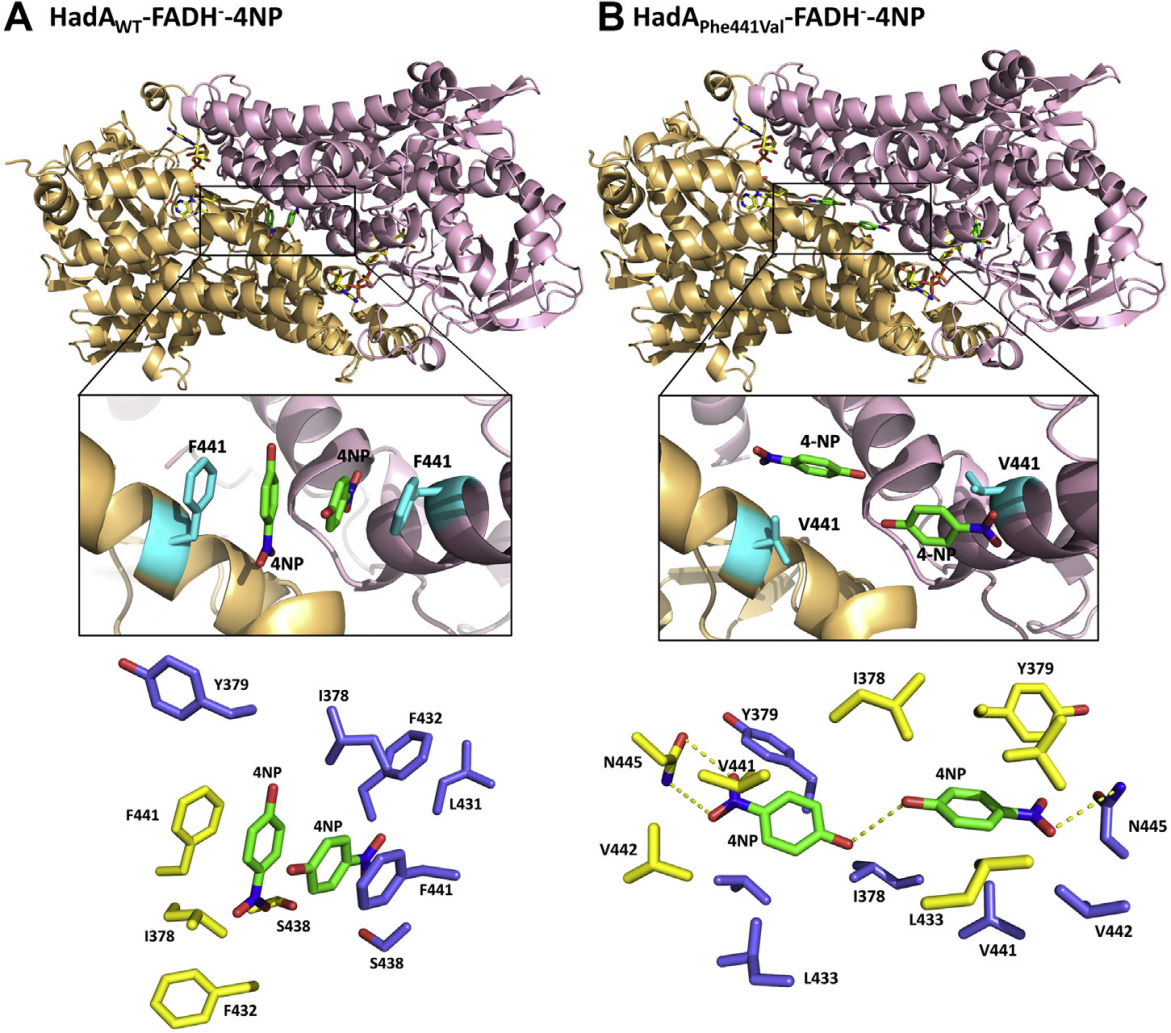
**Intermolecular binding of 4-nitrophenol at the interface of HadA dimer.***A*, a quadruple π-stacking, an unusual π-π-π-π interaction formed by two 4-ntirophenol (4NP) substrates sandwiched with two Phe441 residues of the HadA_WT_ dimer observed in the HadA_WT_–FADH^–^–4NP structure. *B*, disruption of a quadruple π-stacking in the HadA_Phe441Val_–FADH^–^–4NPstructure. The *lower panel* shows side chains of residues from subunit C (*yellow*) and subunit D (*blue*) residing within 4-Å distances from the two 4NP molecules bound in the cavity of the subunit interface.

Apart from electron density of 4NP in the active site, we observed additional electron density of two 4NP molecules bound at the dimer interface between subunits C and D (Fig. 8, *A* and Fig. S8*A*). The area around this region consists of many hydrophobic side chains forming a “hydrophobic cage” that captures two 4NP molecules. The aromatic moieties of the 4NP molecules are sandwiched with the side chains of Phe441 from subunits C and D to perfectly form face-to-face π-π-π-π or quadruple π-stacking (Phe441_C_ = 4NP = 4NP = Phe441_D_) interactions (Fig. 8*A*). This intermolecular hydrophobic interaction is not crucial for assembly of the four subunits because apo-HadA_WT_ can efficiently form a tetramer in the absence of 4NP (16). As Phe441 and the quadruple π-stacking interaction are located at the α15 helix near the FADH^–^-binding site, we hypothesize that this interaction may be the cause of the dead-end complex formation seen for HadA, which hinders procession to the productive pathway when the substrate first binds to the enzyme prior to flavin binding.

### Disruption of quadruple π-stacking in the HadA_Phe441Val_ variant

To further investigate the role of Phe441 and 4NP binding in substrate inhibition, the quadruple π-stacking of 4NP and Phe441 were disrupted by mutating phenylalanine to aliphatic residues including HadA_Phe441Val_, HadA_Phe441Leu_, and HadA_Phe441Ile_. The three-dimensional structure of HadA_Phe441Val_−FADH^–^−4NP was also solved at a 2.3-Å resolution (PDB code: 7E8Q) using a similar approach as used for the structure of HadA_WT_−FADH^–^−4NP (Fig. S9). Data and refinement statistics of this variant three-dimensional structure are listed in Table 1. The overall structure of the ternary complex of HadA_Phe441Val_ is very similar to that of HadA_WT_ (RMSD of 0.143) without any significant changes in the active site area (Fig. S10). Electron density of two 4NP molecules are found in the same hydrophobic tunnel at the interface between the two subunits of the HadA_Phe441Val_ dimer; however, the orientation of the two 4NPs is altered by ∼90° from those observed in HadA_WT_ (Fig. S8*B*). Thus, π-π interaction cannot occur in the variant. The two 4NPs bind *via* van der Waals interactions with nearby residues on the α15 helix with a nitro group (-NO_2_) of 4NP hydrogen bonded with a carboxamide side chain of Asn445 (Fig. 8*B*).

### No dead-end complex formation in HadA_Phe441_ variants

**Figure 9:**
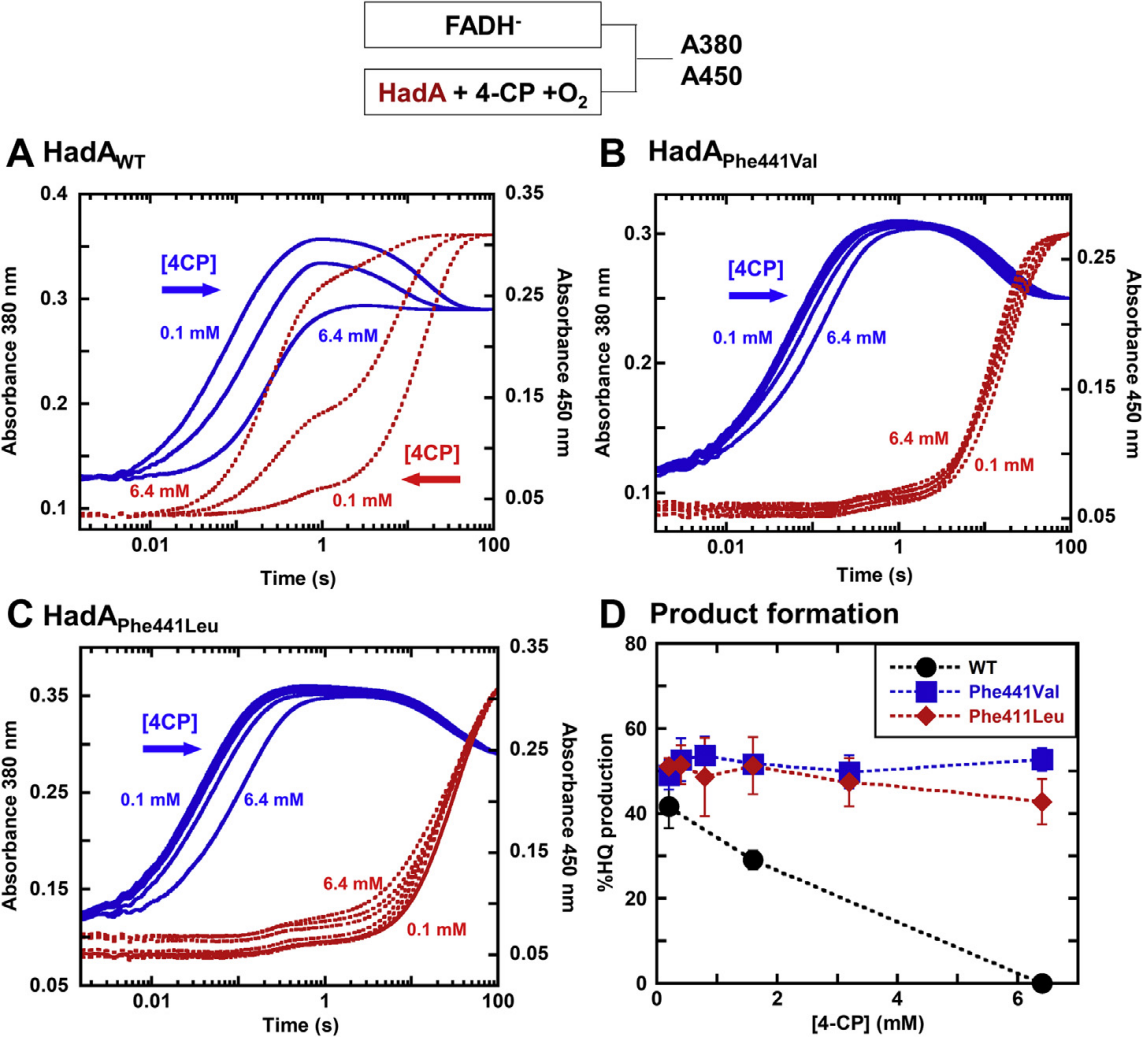
**Regulation of dead-end complex formation by Phe441**. An anaerobic solution of FADH^–^ (25 μM) was rapidly mixed with an air saturated (*A*) HadA_WT_, (*B*) HadA_Phe441Val_, or (*C*) HadA_Phe441Leu_ (75 μM) that was preincubated with 4CP (0.1–6.4 mM) in 20 mM Hepes pH 7.5. Absorption changes at wavelengths 380 (*blue solid line*) and 450 nm (*red dash line*) were monitored to detect formation of the C4a-hydroperoxy-FAD intermediate and oxidized FAD, respectively. C4a-hydroperoxy-FAD absorbs mainly at 380 nm, whereas oxidized FAD absorbs at both wavelengths. *D*, HQ product formation at various concentrations of 4-chlorophenol (4CP) catalyzed by HadA_WT_ (*black circle*), HadA_Phe441Val_ (*blue square*), and HadA_Phe441Leu_ (*red diamond*).

We then further tested whether substrate inhibition by the formation of a dead-end complex still exists in these variants by preincubating the enzymes (HadA_Phe441Val_ and HadA_Phe441Leu_) with various concentrations of 4CP (0.1–6.4 mM) for 5 min to ensure complete formation of the dead-end complex (*k*_*on*_ of 25 M^−1^ s^−1^ for HadA_WT_) (7). The solutions were then rapidly mixed with oxygen to monitor their ability to form C4a-hydroperoxy-FAD using stopped-flow experiments as previously explored in the HadA_WT_ (7). For the reaction of HadA_WT_ (Fig. 9, *A*), increasing the 4CP concentration significantly altered the kinetics of the reaction, as monitored at both *A*_380_ and *A*_450_. Incubation with high concentrations of 4CP showed that the amplitude of *A*_380_ at 1 s was decreased concomitantly with an increase in amplitude of *A*_450_, observed with t_1/2_ ∼ 0.2 s. At the highest concentration of 4CP, the kinetics of the reaction monitored at both *A*_380_ and *A*_450_ were nearly identical, indicating that these changes were due to the formation of oxidized FAD directly without formation of C4a-hydroperoxy-FAD.

In contrast, the signals and amplitude of both *A*_380_ and *A*_450_ in both HadA_Phe441Val_ and HadA_Phe441Leu_ were independent of 4CP concentrations under the same conditions as HadA_WT_ (Fig. 9, *B* and *C*), indicating that the binding of 4CP does not prevent C4a-hydroperoxy-FAD formation. In both variants, the rates of C4a-hydroperoxy-FAD formation were slightly slower at a 4CP concentration of 6.4 mM (blue bold line), whereas the rate of FAD oxidation at the final phase was almost constant (red dashed line). Kinetic traces of the HadA_Phe441Ile_ reactions also showed a similar trend (Fig. S11). All data suggest that no substrate inhibition *via* dead-end complex formation was observed in these HadA_Phe441_ variants, which are void of the quadruple π-stacking complex at the subunit interface.

Hydroquinone (HQ) product from the reactions of HadA_Phe441Val_ and HadA_Phe441Leu_ was analyzed and found to be constant over a range of 4CP concentrations of 0.1 to 6.4 mM 4CP without any signs of substrate inhibition (Fig. 9*D*). These data were significantly different from the reaction of HadA_WT_. These results agreed well with the stopped-flow experiments discussed above in which no substrate inhibition was observed from the behavior of C4a-hydroperoxy-FAD formation. Altogether, the data indicate that, for HadA_Phe441_ variants in which the π-π-π-π complex at the subunit interface is interrupted, no dead-end complex or substrate inhibition could be observed. These enzymes should be able to perform superior catalysis than the HadA_WT_ because they can avoid being trapped in the dead-end enzyme–substrate complex.

### Protein rigidity in HadA_WT_ caused by binding of 4NP

To understand how HadA_WT_ is affected by the binding of 4NP at the subunit interface, we performed thermofluor assays to determine the melting temperatures (*T*_*m*_) of HadA_WT_ and HadA_Phe441_ variants in the presence and absence of 4NP. The *T*_*m*_ values are summarized in Table 2 with melting curves of all enzymes shown in Figure S12. The results showed that the *T*_*m*_ values of the apo forms of HadA_WT_ and the three HadA_Phe441_ variants were similar (∼48 °C). The data indicate that mutation of Phe441 did not alter the structural stability of the apoenzyme. However, the *T*_*m*_ value of HadA_WT_–4NP (in which a quadruple π-stacking exists) was 52 °C which is 4.2 °C higher than that of apo-HadA_WT_. Of interest, the complexes of HadA_Phe441_ variants and 4NP (in which 4NP can bind but the quadruple π-stacking is absent) showed increased *T*_*m*_ values (Δ*T*_*m*_) of only around 1.5 to 2.1 °C. Therefore, the *T*_*m*_ results imply that, upon 4NP binding at the subunit interface, especially with the quadruple π-stacking found in the HadA_WT_, the quaternary structures of HadA are likely more rigid than the apoenzyme forms. This is indicated by the increase in *T*_*m*_ values. The rigidity of the HadA_WT_–4NP complex likely obstructs conformational flexibility particularly at helix α15, leading to the substrate inhibition phenomenon.

**Table 2:** Melting temperature (*T*_*m*_) of HadA_WT_ and HadA_Phe441_ variants

HadA variants	Melting temperature, *T*_*m*_ (°C)		*ΔT*_*m*_ (°C)
	Apoenzyme	With 4NP	
HadA_WT_	48.1 ± 0.9	52.3 ± 0.6	4.2 ± 0.5
HadA_Phe441Val_	47.3 ± 0.7	49.3 ± 0.8	2.1 ± 0.4
HadA_Phe441Leu_	48.2 ± 0.7	49.6 ± 1.1	1.5 ± 0.5
HadA_Phe441Ile_	48.9 ± 0.5	50.5 ± 2.2	1.7 ± 0.9

### Substrate inhibition mechanism in HadA

The data in Figure 9 indicate that HadA_Phe441_ variants are productive over a wide range of 4CP concentrations without showing significant substrate inhibition. The difference between the structures of HadA_WT_ and HadA_Phe441Val_ around the subunit interface areas (Fig. 8) and *T*_*m*_ values (Table 2) clearly explain how the dead-end complex is stabilized in HadA_WT_ but not HadA_Phe441Val_. The unusual face-to-face quadruple π-stacking interactions of four aromatic moieties is the site of “dead-end” complex stabilization, which prevents the formation of C4a-hydroperoxy-FAD, causing inactivation of HadA_WT_. As the distance between the C1 position of 4NP in the productive and the dead-end binding sites is ∼20.8 Å apart (Fig. S13), the inhibition is not caused by direct blocking of 4NP binding to the active site. We propose that the binding of 4NP at the subunit interface may trigger a conformational change in the protein, especially at the α15 helix of the HadA structure, obstructing the proper catalytic site or rigidifying the protein such that it loses its flexibility to properly assume the dynamic changes required for progression of the reaction.

We then searched among enzymes in the same family as HadA to identify whether this feature is prevalent in other systems. The analysis showed that the presence of phenylalanine at the subunit interface is conserved in HadA, *Re*TcpA, *Cn*TcpA, TftD, and HnpA dehalogenases, whereas for other enzymes including CphC-I, DcmB1, NpsA1, NpcA, NpdA2, PheA1, *Tt*HpaB, and *Ec*HpaB, a glutamate is conserved at this position (Fig. 6 and Fig. S4). At the subunit interface of TftD’s structure (PDB: 3HWC) (17), two phenylalanines identified have the potential to form π-π-π-π interactions with a phenolic substrate similar to HadA_WT_. In contrast, the negatively charged glutamate in *Ec*HpaB interface (PDB: 6EB0) does not allow the dead-end complex formation by the quadruple π-stacking (21, 37) (Fig. S14). We propose that this unusual quadruple π-stacking protein–ligand interaction is a distinct property specifically found in HadA_WT_ and closely related enzymes.

### The improvement of HadA catalysis by HadA_Phe441_ variants

**Figure 10:**
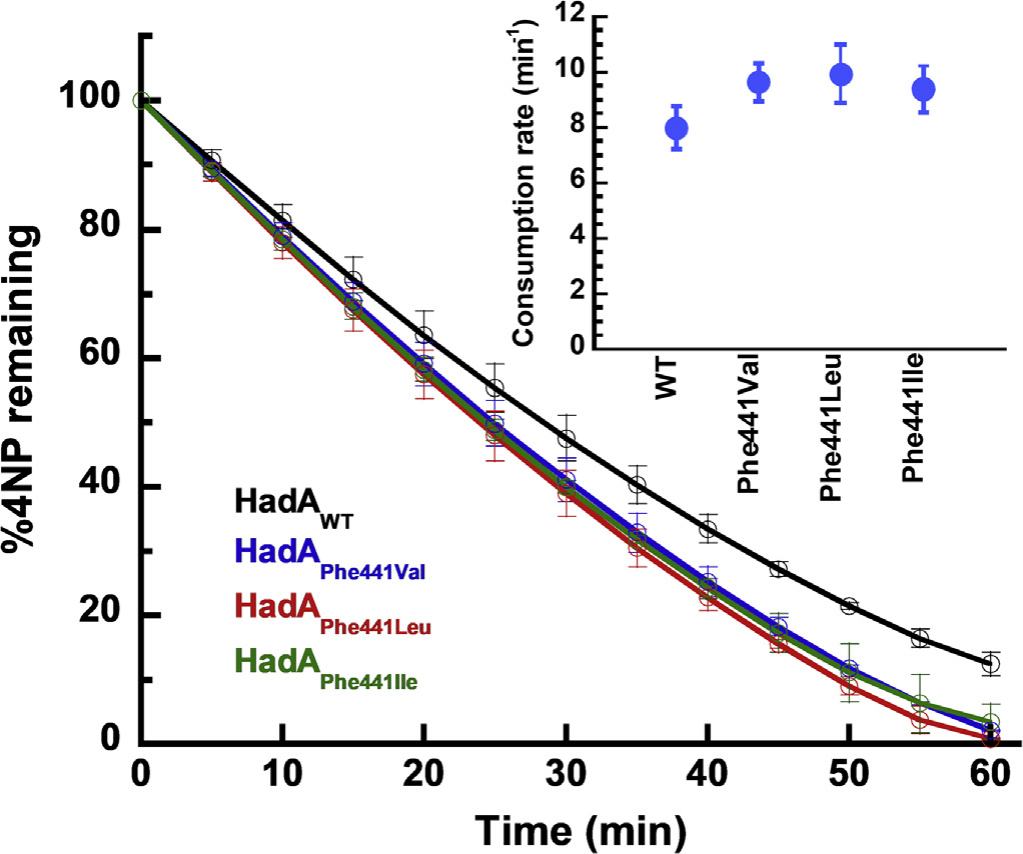
**Efficiency of 4-nitrophenol utilization by HadA**_**Phe441**_**variants.** Multiple turnover reactions consisting of 4NP (100 μM), Glc-6-P (1 mM), Glc-6-PD (0.5 unit/ml), NAD^+^ (5 μM), HadX (1 μM), and HadA_variants_ (0.1 μM) in 20 mM Hepes pH 7.5 were carried out. The molar ratio of 4NP:HadA used is 1000:1. Plots comparing the percentage of 4NP remaining in the denitrification reactions of HadA_WT_ (*black line*), HadA_Phe441Val_ (*blue line*), HadA_Phe441Leu_ (*green line*), and HadA_Phe441Ile_ (*red line*) *versus* time. *Inset* is a plot of rates of 4NP consumption by various types of HadA. 4NP, 4-nitrophenol.

We further explored whether this understanding of the structural features causing substrate inhibition and dead-end complex formation in HadA can be used to improve HadA biocatalysis and its applications. Reactions of HadA_Phe441_ variants and HadA_WT_ were carried out to compare their abilities to detoxify 4NP. The molar ratio between 4NP and HadA was set as 1000:1 to attain conditions in which 4NP significantly inhibits the reaction of HadA_WT_. The results showed that multiple turnover reactions of HadA_Phe441_ variants were more superior than that of HadA_WT_. Within 1 h, the HadA_Phe441_ variants could convert 4NP into BQ at a conversion rate of 100%, whereas only 90% of the 4NP was depleted in the HadA_WT_ reaction (Fig. 10). The rates of 4NP conversion by HadA_Phe441Val_, HadA_Phe441Leu_, and HadA_Phe441Ile_ were 21 ± 8%, 24 ± 13%, and 17 ± 10% greater than that of HadA_WT_, respectively (*p*-value < 0.05) (Fig. 10, *inset*). The data indicate that alteration of Phe441, which removes the quadruple π-stacking interactions and, thus, dead-end complex formation ability, indeed improved biocatalysis by HadA.

## Discussion

The work herein reports the first full package structure of the group D flavin-dependent monooxygenase (HadA) that catalyzes dehalogenation/denitration in addition to hydroxylation. The ternary complex obtained clearly identified important interactions, which were also confirmed by site-directed mutagenesis and stopped-flow experiments. The structural analysis also revealed another binding site of 4NP, which prevents C4a-hydroperoxy-FAD formation and results in the formation of a dead-end complex. Construction of variants in which their 4NP inhibition site was perturbed resulted in variants with improved catalytic activities because the substrate inhibition was abolished.

The tetrameric quaternary structure of HadA monooxygenase co-complexed with FADH^–^ and 4NP is similar to that of apo-HadA, apo-*Re*TcpA, and apo-TftD (15, 16, 17), which share an acyl-CoA dehydrogenase fold like other enzymes in the group D two-component flavin-dependent monooxygenases, including C_2_-FMNH^–^-HPA, *Tt*HpaB-FAD-HPA, and *Ec*HpaB-HPA, previously reported (10, 11, 14, 21, 22, 23) (Fig. S15). These data indicate that the binding of ligands does not affect oligomerization of HadA_WT_ because all forms of the apo or ligand-bound enzymes are tetramers. The FADH^–^-binding region in the HadA active site is also similar to those of group D FADH^–^-utilizing monooxygenases. Binding of FADH^–^ is involved in positioning of the flavin-binding loop (Fig. 2*B*), particularly at residues 157 to 170 because the loop interacts with the adenosine diphosphate moiety of the bound FADH^–^. The flavin-binding loop does not interact closely with the nearby subunit since the distances between the closest regions of the flavin loop (residues 157–160) and the nearby subunit residues (residues 410–415) are longer than 3.5 Å. Specific functions of amino acid residues surrounding FADH^–^ investigated by site-directed mutagenesis, transient kinetics, and product analysis indicate that the bound isoalloxazine ring is stabilized by hydrogen bonding interactions with conserved amino acids such as threonine (Thr193), aspartate (Asp254), and three arginine (Arg101, Arg233, and Arg439) residues. These structural features for FADH^–^ binding are also universal among other two-component monooxygenases in the same family.

On the other hand, the binding pockets of phenol compounds are varied in these enzymes depending on the type of substrates they employ. Since most of their substrates are aromatic compounds, these enzymes including HadA have hydrophobic pockets lined with different hydrophobic amino acid residues. For a single-component flavin-dependent pentachlorophenol hydroxylase (PcpB) from *Sphingobium chlorophenolicum* (member of class A flavin-dependent monooxygenases), which can degrade highly hydrophobic substrates such as pentachlorophenol (43), its binding pocket is rather different from those of the HadA family. The modeled active site of PcpB consists of four phenylalanine residues to maintain hydrophobic interactions with substrate (44, 45). For class D two-component monooxygenases that catalyze hydroxylation, only two X-ray structures are available for the enzyme co-complexed with the flavin and aromatic substrate, i.e., *Tt*HpaB-FAD-4HPA from *T. thermophilus* HB8 (PDB code: 2YYJ) (22) and C_2_-FMNH^–^-4HPA from *A. baumannii* (PDB code: 2JBT) (23) with RMS of C_α_ alignment of 1.773 and 10.105, respectively, are available. Although the overall foldings of these enzymes are similar to that of HadA, the architecture inside their active sites is quite different (Fig. S15) because HadA catalyzes hydroxylation with dehalogenation/denitration at position C4 of phenol substrates, whereas *Tt*HpaB and C_2_ catalyze only hydroxylation at the *ortho*-position of *p*-hydroxyphenylacetate.

The binding of reduced flavins seems to be crucial for phenolic substrate binding. We could not crystalize a binary complex of HadA–4NP or HadA–4CP in the absence of FADH^–^. X-ray structures of similar binary complexes also could not be obtained for C_2_ and *Tt*HpaB (22, 23). Kinetic investigation of *p*-hydroxyphenylacetate hydroxylases from *A. baumannii* and *Pseudomonas aeruginosa* indicates that the substrate 4HPA only binds after the flavin binding (24, 46, 47). These imply that the bound flavin may create a recognition site for a phenolic substrate. For HadA_WT_, although a phenolic substrate can bind, it leads to the formation of the dead-end complex. Under physiological conditions, as the rate of FADH^–^ binding to HadA_WT_ is faster than 4CP binding by four orders of magnitude (7), these properties would help prevent the enzyme from getting trapped in the dead-end complex.

The structure reported here also explains the root cause of substrate inhibition, which is a common obstacle in biocatalytic applications. The rigidity of a perfect face-to-face quadruple π-stacking complex formed from four aromatic moieties (two aromatics from phenylalanine side chains and two aromatics from substrates) may interfere with the FADH^–^-binding site. This conclusion was confirmed by single point mutations at Phe441 that disrupted this quadruple π-stacking complex, resulting in enzyme variants with improved biocatalytic activities. Usually, the π-π interaction is observed in protein–protein or protein–ligand interactions to facilitate protein function. For example, binding of aromatic substrate in the active site of PcpB from *S. chlorophenolicum* (44, 45), nicotine oxidoreductase (NicA2) from *Pseudomonas putida* (48), and also HadA_WT_ itself is mediated by π-π interactions. The π-π interactions could also be applied in various biological applications such as in the development of molecular receptors, in the design of controlled drug release systems, and in the fabrication of biosensors (49, 50).

The mechanism of dead-end complex formation in HadA shares some common features with those of other flavin-dependent monooxygenases such as bacterial luciferase and halogenase. Bacterial luciferase from *Vibrio harveyi* belongs to class C flavin-dependent monooxygenases. Binding of an aldehyde substrate to apo-luciferase blocks the binding of FMNH^–^ resulting in a decrease in the light emission reaction (18, 19, 20). The equilibrium binding experiments revealed two aldehyde-binding sites at each α and β2 subunit. The stronger-binding aldehyde binds to the active site and acts as a substrate, whereas the weaker-binding aldehyde binds to an inhibitory pocket (51). For flavin-dependent halogenases, RebH from *Lechevalieria aerocolonigenes* and Thal from *Streptomyces albogriseolus*, which are class F monooxygenases, stopped-flow investigation showed that preformation of the RebH–FADH^–^ or Thal–FADH^–^ complexes prior to binding of a tryptophan substrate results in abolishment of C4a-hydroperoxyflavin formation (52, 53). Molecular dynamics simulations of Thal–FADH^–^ showed rearrangement of water molecules upon prolonged incubation of Thal and FADH^–^, which may affect the protonation status of FADH^–^ and stabilization of C4a-hydroperoxy-FAD (52). For class A NicA2 from *P. putida*, a pocket ∼12 Å away from the active site can accommodate nicotine binding, which may cause substrate inhibition (48).

As flavin-dependent monooxygenases are attractive for many applications (3, 10, 11, 12, 14, 54), identification of residues important for catalysis provides basic knowledge for future rational design in enzyme engineering. As HadA is useful for many applications including detoxification and biodetection (9), the structural basis reported here should be useful for improving HadA for industrial applications. In the past, engineering of C_2_ resulted in variants useful for biocatalysis. The variant Tyr398Ser of C_2_ catalyzes formation of trihydroxyphenolic acids with higher efficiency than the wild-type enzyme (55), whereas single mutations at either Ser146 or Arg263 resulted in C_2_ variants, which can use *p*-aminophenylacetate and *p*-hydroxyphenylethylamine better than the native substrate 4HPA, respectively (56, 57). Mutation of group C flavin-dependent luciferases can also change their properties, such as alteration of emission color or prolonging light intensity of the product, which is useful for biodetection applications (58).

In conclusion, these in-depth structural insights into the catalysis and inhibition mechanisms of HadA flavin-dependent monooxygenase with additional dehalogenating/denitrating activities provide an important basis for better understanding flavin-dependent monooxygenases and for future enzyme engineering to more effectively implement these enzymes in applications including biodetoxification and biodetection of toxic compounds as well as in biocatalysis.

## Experimental Procedures

### Protein crystallization

HadA was purified for crystallization according to a reported protocol (7). The purified enzyme was stored in 20 mM Hepes pH 7.5 containing 20% (v/v) glycerol and kept in a –80 °C freezer until use. A stock of FAD solution (high concentration) was dissolved in filtered H_2_O. The concentration of FAD was calculated based on the absorption at a wavelength 450 nm using an extinction coefficient ε_450_ of 11.3 mM^−1^ cm^−1^. Reduced FAD (FADH^–^) was prepared by stoichiometric reduction of FAD using sodium dithionite inside an anaerobic glove box. A stock of 4NP at high concentration was prepared by dissolving in dimethyl sulfoxide (Sigma). All components of crystallizing agents were filtered by passage through syringe filters with 0.22-μm pore size before use. All equipment for crystallization were made anaerobic before being used. All experiments using FADH^–^ were performed inside an anaerobic glove box (Belle Technology Ltd) to avoid reoxidation by molecular oxygen.

For crystallization of the HadA–FADH^–^–4NP complex, HadA (0.3 mM), FADH^–^ (12 mM), and 4NP (12 mM) were mixed inside the anaerobic glove box. Concentrations indicated were the final concentrations in the protein complex that was incubated for 2 h to ensure complete complex formation prior to performing crystallization. A solution of the protein complexes (1 μl) was mixed with crystallizing agents (1 μl) using the microbatch technique and a 60-well minitray (1 mm diameter at bottom of each well) covered with 6 ml of baby oil (Johnson; a mixture of mineral oil, olive oil, and vitamin E, PZ Johnson). The protein complex was crystallized at 25 °C in small grids of 0.18 to 0.22 M sodium citrate dehydrate (Sigma), 15% to 17% (w/v) polyethylene glycol 3350 or PEG3350 (Hampton research), 5% (v/v) 2,2,2-trifluoroethanol (Fluka) as an additive, and 0.1 M Bis-Tris propane (Sigma) pH 6.5 as the buffer. Crystals of HadA complexes were harvested by quick dipping in the crystallizing agent containing substrate and 15% (v/v) glycerol as cryoprotectants prior to storage in liquid nitrogen.

### Data collection and structure solving

X-ray diffraction data of HadA complexes were collected at 100 K at a wavelength of 1.54 Å using a D8 Venture single crystal X-ray diffractometer coupled with a PHOTON 100 detector (Bruker). The structure of HadA was determined by molecular replacement using Phaser in the CCP4 suit (59) with a chain A of apo-HadA (PDB code: 6JHM) as a template. Model building and structure refinement were performed using Coot (60) and REFMAC5 (61). The ligand structure was prepared using Hyperchem.

### Site-directed mutagenesis and preparation of HadA variants

Sited-directed mutagenesis was performed using PCR and the *hadA*_*WT*_-pET-11a plasmid as a template. In brief, the solutions (50 μl) of ∼50 ng of *hadA*_*WT*_-pET-11a plasmid, 0.4 μM of both forward and reverse primers listed in Table S1 (Bio Basic Inc), 0.2 mM of dNTPs, and 0.05 U/μl of *Pfu* DNA polymerase (Thermo Scientific) or 0.02 U/μl Q5 High-fidelity DNA polymerase (New England Biolabs Inc) in 1× buffer were mixed. The PCR condition was hot started at 95 °C for 5 min followed by 16 cycles of denaturation at 95 °C for 45 s, annealing at 60 °C for 1 min, and extension at 72 °C for 15 min. The reaction was kept at 72 °C for 18 min in the final cycle. PCR products were treated with *Dpn*I (New England Biolabs Inc) to remove the *hadA*_*WT*_-pET-11a template. Mutated plasmids were transformed into *E. coli* XL1–Blue and grown on LB agar plate containing ampicillin (50 μg/ml) at 37 °C. Colonies were selected, grown, and purified to obtain plasmid DNA. Selected plasmids were sequenced to confirm accuracy of the mutation (Macrogen Inc or U2Bio). HadA variants were purified using the same approach used for HadA_WT_ (7).

### Activity assay of HadA

Activity of HadA variants was analyzed using a coupled enzymatic system consisting of HadA and reduced flavin regenerating enzymes including glucose-6-phosphate dehydrogenase (Glc-6-PD) and HadX flavin reductase (6). The reaction consisting of 4NP (50 μM), glucose-6-phosphate (Glc-6-P, 1 mM), Glc-6-PD (0.5 unit/ml), NAD^+^ (5 μM), HadX (1 μM), HadA variants (10 μM) was carried out in 20 mM Hepes pH 7.5. The absorption change at a wavelength of 400 nm was monitored to measure the loss of 4NP using a UV-visible spectrophotometer Cary 60 (Agilent Technology).

### Transient kinetics experiments of HadA variants

Rapid kinetics experiments of HadA variants were performed using similar methods previously used for HadA_WT_ (7). A binary complex of HadA_variants_:FADH^–^ (75 μM:25 μM) was prepared by reduction of HadA_variants_:FAD with a slight excess amount of sodium dithionite inside the anaerobic glove box before placing them in a tightly capped anaerobic syringe to avoid auto-oxidation by oxygen. A solution of anaerobic HadA_variants_:FADH^–^ was mixed with an aerobic solution 4CP (0.5 mM with 0.13 mM oxygen) on a single-mixing stopped-flow spectrophotometer model SF-61SX (TgK Scientific, Bradford-on-Avon) in which the flow parts were made anaerobic by flushing with an oxygen scrubbing solution overnight (25). Reagent concentrations are expressed as the final concentrations after mixing. Absorption changes at wavelengths 380 nm (*A*_380_) and 450 nm (*A*_450_) were monitored to indicate the formation of C4a-hydroperoxy-FAD and oxidized FAD, respectively. To monitor dead-end complex formation, the aerobic enzyme solution was mixed with a substrate and incubated for certain periods before mixing with FADH^–^ to initiate the formation of C4a-hydroperoxy-FAD similar to the approach used in the HadA_WT_ reaction previously reported (7). 4NP was not used in this experiment because the compound has high absorbance at 400 nm, interfering with observation of the kinetics of flavin oxidation.

### HQ production analysis

Samples from single turnover reactions were prepared and collected for analysis using the same methods described (7). The BQ product was reduced to HQ, which is more stable by adding ascorbic acid (0.5 mM). Production of HQ product was analyzed on an HPLC 1100 series (Agilent Technologies) equipped with a UV-visible diode array detector. A Nova-Pak (Waters) C18 reverse phase column with a 4-μm particle size and a 3.9 × 150-mm column size was used as the stationary phase. A gradient of H_2_O/methanol containing 0.1% formic acid (10%–70% methanol) was used as a mobile phase. HQ was detected at a wavelength of 289 nm at a retention time of 3.9 min.

### Molecular docking

Binding of 4NP to the active sites of HadA variants was investigated by AutoDock Vina (62). Structures of HadA variants were prepared by mutagenesis tools using the PyMol software with appropriate rotamers. The configurations of nonpolar moieties were assigned using AutoDock Tools 1.5.7 with the cubic set at 8 to 10 Å and the coordinate of C1 of 4NP in the HadA_WT_ structure as a center with an energy range of four and exhaustiveness of eight. The affinity energy was collected from the lowest-energy conformation aligned in the correct orientation.

### Thermofluor assays

A solution of HadA_WT_ or HadA_Phe441_ variants (10 μM) was mixed with 5× SYPRO orange dye (Sigma) in 20 mM Hepes pH 7.5 in the absence or presence of 4NP (2 mM). In the presence of 4NP, enzymes and substrate were preincubated for 1 h prior to mixing with SYPRO orange dye in 20 mM Hepes pH 7.5. A real-time PCR instrument model Rotor Gene Q (Qiagen) was used to monitor fluorescence changes due to protein unfolding to determine the melting temperature (*T*_*m*_) of HadA (16). The program’s temperature was gradually increased from 35 °C to 95 °C with 0.5 °C/min increment.

### Statistical analysis

Data from at least three replications were used for calculating means ± standard deviations (SD). Statistical significance analysis was performed using the independent *t* test from Statistical Package for the Social Sciences Program (IBM).

## Data availability

All data are included in the article and supporting information. Data of X-ray structures are available at Protein Data Bank under PDB codes indicated.

## Supporting information

This article contains supporting information.

## Conflict of interest

The authors declare that they have no conflict of interest with the contents of this article.
