# Supplementary material for: Structural insights into a flavin-dependent dehalogenase HadA explain catalysis and substrate inhibition via quadruple π-stacking
Source: J Biol Chem. 2021 Jul 10;297(2):100952. doi: 10.1016/j.jbc.2021.100952 (PMC8342789; doi:10.1016/j.jbc.2021.100952)
Supplement: Figures S1–S15 and Table S1 [file mmc1.pdf]

Supporting Information

Structural Insights into a Flavin-dependent Dehalogenase HadA Explain Catalysis and Substrate Inhibition via Quadruple π-stacking

Panu Pimviriyakul[1], Aritsara Jaruwat[2], Penchit Chitnumsub[2,*] and Pimchai Chaiyen[3,*]

[1]Department of Biochemistry, Faculty of Science, Kasetsart University, Chatuchak, Bangkok, 10900, Thailand.
[2]National Center for Genetic Engineering and Biotechnology, 113 Thailand Science Park, Phahonyothin Road, Pathumthani, 12120, Thailand.
[3]School of Biomolecular Science and Engineering, Vidyasirimedhi Institute of Science and Technology (VISTEC), Wangchan Valley, Rayong, 21210, Thailand.

*Running title: Structures of a HadA Flavin-Dependent Dehalogenase

*Corresponding author
E–mail: pimchai.chaiyen@vistec.ac.th; penchit@biotech.or.th

Table S1. List of primers for site-directed mutagenesis of HadA variants.

Variants	Direction	Primer Sequence	Expression in soluble form
Thr193Ala	Forward<br>Reverse	5’ AAGGCCATCGGCGCCGGCGTGGCCTTTG 3’<br>5’ CAAAGGCCACGCCGGCGCCGATGGCCTT 3’	Yes
Thr193Val	Forward<br>Reverse	5’ AAGGCCATCGGCGTGGGCGTGGCCTTTG 3’<br>5’ CAAAGGCCACGCCCACGCCGATGGCCTT 3’	Yes
Thr193Ser	Forward<br>Reverse	5’ AAGGCCATCGGCTCGGGCGTGGCCTTTG 3’<br>5’ CAAAGGCCACGCCCGAGCCGATGGCCTT 3’	Yes
Thr193Cys	Forward<br>Reverse	5’ AAGGCCATCGGCTGCGGCGTGGCCTTTG 3’<br>5’ CAAAGGCCACGCCGCAGCCGATGGCCTT 3’	No
Phe206Ala	Forward<br>Reverse	5’ CACATCGGCGTGGCCTTCCGCCCCGGT 3’<br>5’ ACCGGGGCGGAAGGCCACGCCGATGTG 3’	No
Phe206Val	Forward<br>Reverse	5’ CACATCGGCGTGGTCTTCCGCCCCGGT 3’<br>5’ ACCGGGGCGGAAGACCACGCCGATGTG 3’	Yes
Phe206Leu	Forward<br>Reverse	5’ CACATCGGCGTGCTCTTCCGCCCCGGT 3’<br>5’ ACCGGGGCGGAAGAGCACGCCGATGTG 3’	Yes
Phe206Ile	Forward<br>Reverse	5’ CACATCGGCGTGATCTTCCGCCCCGGT 3’<br>5’ ACCGGGGCGGAAGATCACGCCGATGTG 3’	Yes
Asp254Ala	Forward<br>Reverse	5’ GCGACGAACTCGCAGGCATGACCGT 3’<br>5’ ACGGTCATGCCTGCGAGTTCGTCGC 3’	Yes
Asp254Asn	Forward<br>Reverse	5’ GCGACGAACTCAACGGCATGACCGT 3’<br>5’ ACGGTCATGCCTTGGAGTTCGTCGC 3’	Yes
Phe286Ala	Forward<br>Reverse	5’ CCGCAGCGCGTGGCCGACTGGCTGCAC 3’<br>5’ GTGCAGCCAGTCGGCCACGCGCTGCGG 3’	Yes
Phe286Val	Forward<br>Reverse	5’ CCGCAGCGCGTGGTCGACTGGCTGCAC 3’<br>5’ GTGCAGCCAGTCGACCACGCGCTGCGG 3’	Yes
Phe286Leu	Forward<br>Reverse	5’ CCGCAGCGCGTGCTCGACTGGCTGCAC 3’<br>5’ GTGCAGCCAGTCGAGCACGCGCTGCGG 3’	Yes

Variants	Direction	Primer Sequence	Expression in soluble form
Phe286Ile	Forward<br>Reverse	5’ CCGCAGCGCGTGATCGACTGGCTGCAC 3’<br>5’ GTGCAGCCAGTCGATCACGCGCTGCGG 3’	Yes
His290Ala	Forward<br>Reverse	5’ GTTCGACTGGCTGGCCTACCACGCGCTG 3’<br>5’ CAGCGCGTGGTAGGCCAGCCAGTCGAAC 3’	Yes
His290Lys	Forward<br>Reverse	5’ GTTCGACTGGCTGAAGTACCACGCGCTG 3’<br>5’ CAGCGCGTGGTACTTCAGCCAGTCGAAC 3’	No
His290Arg	Forward<br>Reverse	5’ GTTCGACTGGCTGCGCTACCACGCGCTG 3’<br>5’ CAGCGCGTGGTAGCGCAGCCAGTCGAAC 3’	No
His290Val	Forward<br>Reverse	5’ GTTCGACTGGCTGGTGTACCACGCGCTG 3’<br>5’ CAGCGCGTGGTACACCAGCCAGTCGAAC 3’	No
His290Gln	Forward<br>Reverse	5’ GTTCGACTGGCTGCAGTACCACGCGCTG 3’<br>5’ CAGCGCGTGGTACTGCAGCCAGTCGAAC 3’	No
His290Asn	Forward<br>Reverse	5’ GTTCGACTGGCTGAACTACCACGCGCTG 3’<br>5’ CAGCGCGTGGTAGTTCAGCCAGTCGAAC 3’	Yes
His290Cys	Forward<br>Reverse	5’ GTTCGACTGGCTGTGCTACCACGCGCTG 3’<br>5’ CAGCGCGTGGTAGCACAGCCAGTCGAAC 3’	Yes
His290Asp	Forward<br>Reverse	5’ GTTCGACTGGCTGGACTACCACGCGCTG 3’<br>5’ CAGCGCGTGGTAGCACAGCCAGTCGAAC 3’	No
His290Glu	Forward<br>Reverse	5’ GTTCGACTGGCTGGAGTACCACGCGCTG 3’<br>5’ CAGCGCGTGGTACTCCAGCCAGTCGAAC 3’	No
Phe441Val	Forward<br>Reverse	5’ CAGCCGCCTGGTCGTGTTCGAGA 3’<br>5’ TCTCGAACACGACCAGGCGGCTG 3’	Yes
Phe441Leu	Forward<br>Reverse	5’ CAGCCGCCTGCTCGTGTTCGAGA 3’<br>5’ TCTCGAACACGAGCAGGCGGCTG 3’	Yes
Phe441Ile	Forward<br>Reverse	5’ CAGCCGCCTGATCGTGTTCGAGA 3’<br>5’ TCTCGAACACGATCAGGCGGCTG 3’	Yes

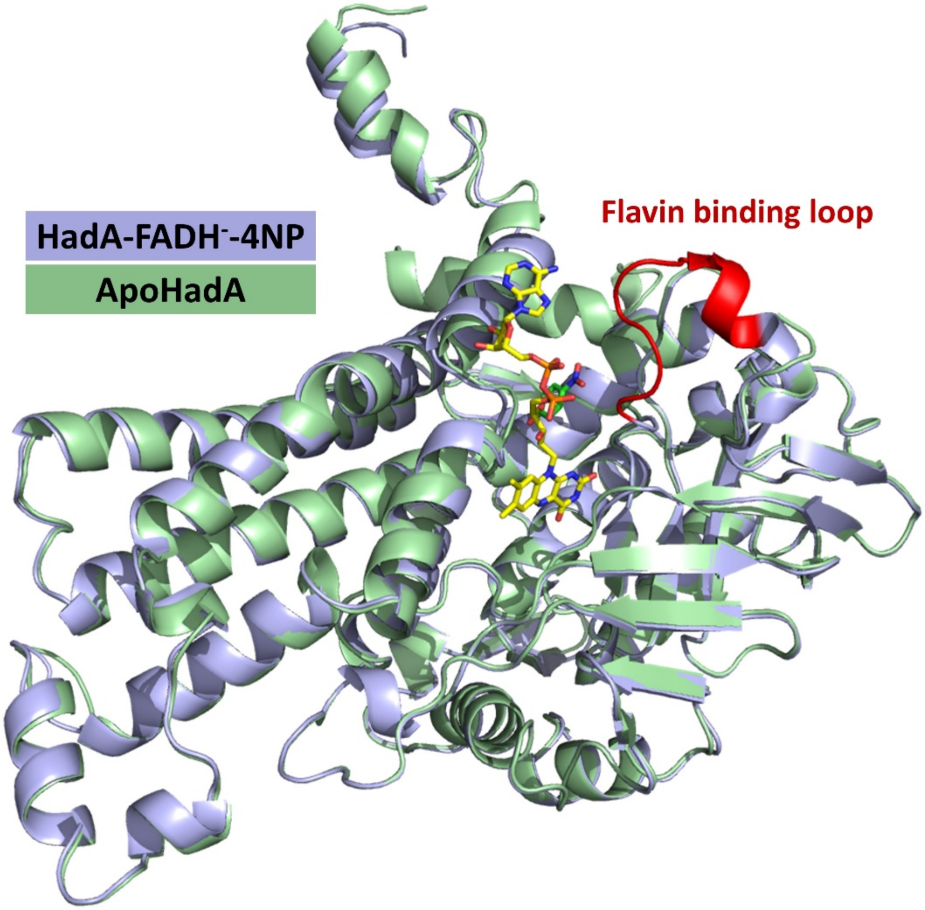

Figure S1. Superimposed structures of apo-HadA$_{WT}$ (pale green) and HadA$_{WT}$-FADH^{-}-4NP complex (pale purple) showing 4NP (in green) and FADH^{-} (in yellow). The superposition was performed by PyMol with C$_{\alpha}$ RMSD of 0.439.

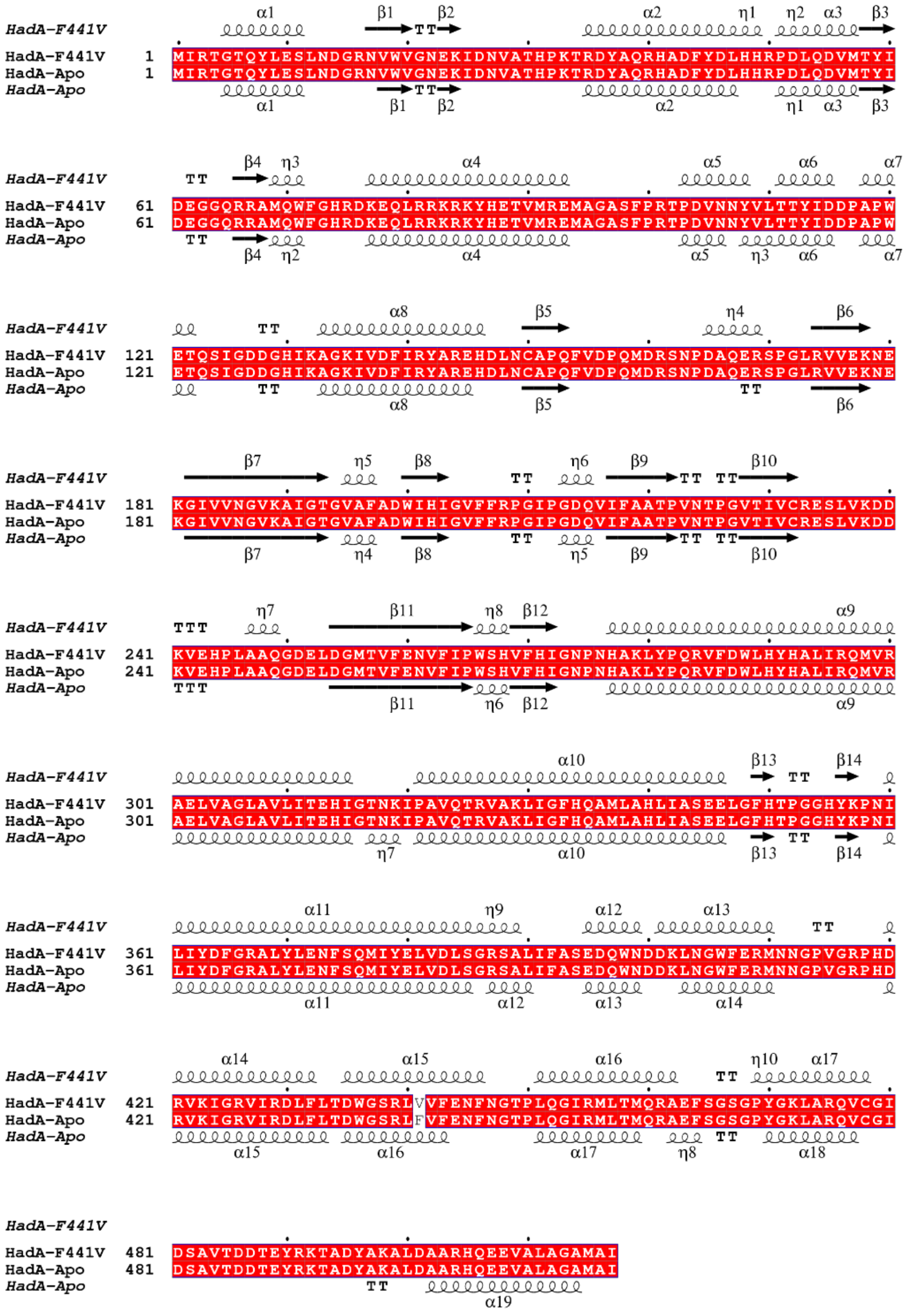

Figure S2. The alignment of secondary structures of the HadA-FADH$^-$-4NP complex and apo-HadA$_{WT}$ is drawn with ESPript.

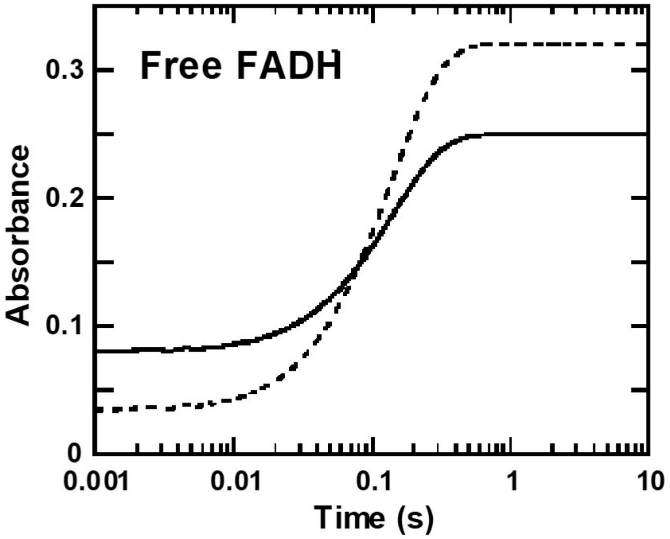

Figure S3. Kinetic data of free FADH⁻ oxidation. Kinetic traces obtained from rapid kinetics of reactions of FADH⁻ (25 μM) mixing with an aerobic (0.13 mM O_2) solution of 20 mM HEPES pH 7.5 in single mixing mode of a stopped-flow apparatus. Absorption changes at wavelengths 380 nm (solid line) and 450 nm (dashed line) have similar kinetics with rate constants of 7.0 ± 0.1 s^{-1}.

```
HadA      -------------------------------------MIRTGTQYLESLNDG-RNVWVGN   22
ReTcpA    -------------------------------------MIRTGKQYLESLNDG-RNVWVGN   22
CnTcpA    -------------------------------------MIRTGKQYLESLNDG-RNVWVGN   22
TftD      --------------------------------------MRTGKQYLESLNDG-RVVWVGN   21
CphCI     --------------------------------------MRTGKEYLESLRDG-RKVYVGG   21
DcmB1     MTTTEIPPTGVDPADSGAPQVNPAADCEANRTKNFATRPMTGDEYISSLQDG-REIWLHG   59
HnpA      -------------------------------------MIRTGKQYLESLNDG-RNVWVGN   22
NpsA1     --------------------------------------MRTGQQYLESLRDG-RQVYVGG   21
NpcA      --------------------------------------MRTGQQYLESLRDG-RQVYVGG   21
NpdA2     --------------------------------------MRTGKEYLESLRDG-RQVYVGG   21
PheA1     -----------------------------------MKDMMNGKEYLESLRDN-RVVYLNG   24
TtHpaB    -------------------------------------MARTGAEYIEALKTRPPNLWYKG   23
EcHpaB    --------------------------MKPEDFRASTQRPFTGEEYLKSLQDG-REIYIYG   33
C2        ---------------------------------------------ME----N-TVLNLDS   10
                                                       :.        :   .

HadA      EKIDNVATHPKTRDYAQRHADFYDLHHRPDLQ---------DVMTYID--EGGQRRAMQW   71
ReTcpA    EKIDNVATHPKTRDYAQRHADFYDLHHRPDLQ---------DVMTFVD--KDGERRTMQW   71
CnTcpA    EKIDNVATHPKTRDYAQRHADFYDLHHRPDLQ---------DVMTFVD--TDGERRTMQW   71
TftD      EKIDNVATHPLTRDYAERVAQFYDLHHRPDLQ---------DVLTFVD--ADGVRRSRQW   70
CphCI     ELIEDVTTHPKTKGYAQAIAEYYDLHLKPENQ---------DLLTFVD--ENGKRESMHW   70
DcmB1     DRVKDVTTHPAFRNPIRMTARLYDALHTGEHV---------DALTVPTDTGNGGVTMPFF   110
HnpA      EKIDNVATHPKTRDYAQRHADFYDLHHRPDLQ---------DVMTFVD--TDGERRTMQW   71
NpsA1     ELIDDVTTHPKTSGYAKAIAEYYDLHLDPEHQ---------DVLTFVD--DDGVRKSMHW   70
NpcA      ELIDDVTTHPKTSGYAKAIAEYYDLHLDPEHQ---------DVLTFVD--DDGVRKSMHW   70
NpdA2     ELIEDVTTHPKTKGYAQAIAEYYDLHLKPENQ---------DLLTFVD--ENGKRESMHW   70
PheA1     EKIDDVTTHPAYENAARSIARMYDALHDEQMG---------KILTTT--TEEGYRTHKFF   73
TtHpaB    EKVEDPTTHPVFRGIVRTMAALYDLQHDPRYR---------EVLTYEE---EGKRHGMSF   71
EcHpaB    ERVKDVTTHPAFRNAAASVAQLYDALHKPEMQ---------DSLCWNTDTGSGGYTHKFF   84
C2        DVI------HACEAIFQPIRLVYTHAQTPDVSGVSMLEKIQQILPQIA--KNAESAEQLR   62
          : :                   *                  . :        .
                                      R101
HadA      FGHRDKEQLRRKRKYHETVMREMAGASFPRTPDVNNYVLTTYIDDP-APWETQSIGDDGH   130
ReTcpA    FGHYDKEQLRRKRKYHETIMREMAGASFPRTPDVNNYVLQTYIDDP-SPWETQTIGAEGK   130
CnTcpA    FGHFDKEQLRRKRKYHETIMREMAGASFPRTPDVNNYVLQTYIDDP-SPWETQTIGAEGK   130
TftD      QDPKDAAGLRVKRKYHETILREIAAGSYGRLPDAHNYTFTTYADDP-EVWEKQSIGAEGR   129
CphCI     FLPRSKEDVIKRRNYADFIFRHFQGGIFTRPPAGMNVVMFTQVDDQ-EPWAENSRFKNGH   129
DcmB1     RTPTSSADLLKERDAIATWAR-MTYGWMGRSPDYKASFLGTLHANK-ELYA---------   159
HnpA      FGHFDKEQLRRKRKYHETIMREMAGASFPRTPDVNNYVLQTYIDDP-SPWETQTIGAEGK   130
NpsA1     FLPRSKADAARRRAYHEFWFRHFQGGIFTRPPAGMHVVMYAQIDDP-EPWGDNAVVAGGR   129
NpcA      FLPRSKADAARRRAYHEFWFRHFQGGIFTRPPAGMHVVMYAQIDDP-EPWGDNAVVAGGR   129
NpdA2     FLPRSKEDVVKRRNYADFIFRHFQGGIFTRPPAGMNVVMFTQVDDQ-EPWAENSRFKNGH   129
PheA1     KEPKNAQDLLEARDAIAQWAK-LSYGFMGRTPDYKASFTAHLKAFA-DYYE---------   122
TtHpaB    LIPKTKEDLKRRGQAYKLWAD-QNLGMMGRSPDYLNAVVMAYAASA-DYFG---------   120
EcHpaB    RVAKSADDLRQQRDAIAEWSR-LSYGWMGHTPDYKAAFGCALGANP-GFYG---------   133
C2        RVPDENIKLLKEIGLHRAFQPKVYGGLEMSLPDFANCIVTLAGACAGTAWAFSLLCTHSH   122
                                   .     *                 :
                                V155Q158R161
HadA      I--KAGKIVDFIRYAREHDLNCAPQFVDPQMDRSNPDAQERSPGLR--VVEKNEKGIVVN   186
ReTcpA    V--KAKNIVDFVNFAKKHDLNCAPQFVDPQMDRSNPDAQQRSPGLR--VIEKNDKGIVVS   186
CnTcpA    I--KAKNIVDFVNFAKQHDLNCAPQFVDPQMDRSNPDAQQRSPGLR--VIEKNDKGIVVS   186
TftD      N--LTQNIHNFLKLLREKDLNCPLNFVDPQTDRSSDAAQARSPNLR--IVEKTDDGIIVN   185
CphCI     R-DLSGNIQRHWDEVTAKDLAVSPMFVDVQYDRGRDDSMAETPMLS--IIEENDEGIVVR   186
DcmB1     --PFQDNAERWYRESQEKVLYWNHAIINPPVDRQLPPDEVGDVFMK--VEKETDAGLIVS   215
HnpA      I--KAKNIVDFVNFAKQHDLNCAPQFVDPQMDRSSPDAQQRSPGLR--VIEKNDKGIIVS   186
NpsA1     TISFADNIRSQWQRVTTDDVALSPMFVDVQFDRGRDDALVETPMLS--IVEQNDQGIVVR   187
NpcA      TISFADNIRSQWQRVTTDDVALSPMFVDVQFDRGRDDALVETPMLS--IVEQNDQGIVVR   187
NpdA2     R-DLSGNIQRHWEEVTSKDLAVSPMFVDVQYDRGRDDAMAETPMLS--IIEENDEGIVVR   186
PheA1     --GFEDNARNWYKKTTKEIPFINHTIINPQVDRSKPLHENKDVFVR--AVKERDDGIIVS   178
TtHpaB    --EFAENVRNYYRYLRDQDLATTHALTNPQVNRARPPSGQPDPYIPVGVVKQTEKGIVVR   178
EcHpaB    --QFEQNARNWYTRIQETGLYFNHAIVNPPIDRHLPTDKVKDVYIK--LEKETDAGIIVS   189
C2        Q------IAMFSKQLQ------------DEIWLKDPDATASSSIAPFGKVEEVEGGIILN   164
                                                    :: : *:::
```

```
                  T193         F206                          R233
HadA        GVKAIGTGVAFADWIHIGVFFRPGIPG---DQVIFAATPVNTPGVTIVCRESLVK-----   238
ReTcpA      GVKAIGTGVAFADWIHIGVFFRPGIPG---DQIIFAATPVNTPGVTIVCRESVVK-----   238
CnTcpA      GVKAVGTGVAFADWIHIGVFFRPGLPG---DQIIFAATPVNTPGVTIVCRESVVK-----   238
TftD        GVKAVGTGIAFGDYMHIGCLYRPGIPG---EQVIFAAIPTNTPGVTVFCRESTVK-----   237
CphCI       GWKAIGTSIPFVNNLLIGNLWRPGQTA---EQTIYAMVPLATPGVSVVARESRAQ---PD   240
DcmB1       GAKVVATGSAITNYNFIAHYGLP--IKK-KQFALICTVPMDAPGVKLICRTSYTEHAAVM   272
HnpA        GVKAIGTGVAFADWIHIGVFFRPGIPG---DQIIFAATPVNTPGVTIVCRESVVK-----   238
NpsA1       GWKAMGTSLPFVNELLVGNLWRPGQTS---DQTVYAIVPVNTPGLSLVCRQSNAT---PD   241
NpcA        GWKAMGTSLPFVNELLVGNLWRPGQTS---DQTVYAIVPVNTPGLSLVCRQSNAT---PD   241
NpdA2       GWKAIGTSIPFVNNLLIGNLWRPGQTA---EQTVYAMVPLATPGVSVVARESRAQ---PD   240
PheA1       GAKMVGTAAALTHYNFVSNYGAQDLGDGDQSHALIFFVPMNAPGVKMISRQSYEQIAKTL   238
TtHpaB      GARMTATFPLADEV---LIFPSTLLQAGSEKYALAFALPTSTPGLHFVCREALV----GG   231
EcHpaB      GAKVVATNSALTHYNMIGFGSAQVMGEN-PDFALMFVAPMDADGVKLISRASYEMVAGAT   248
C2          GDYGWSSGCDHAEYAIVGFNRFDADGN---KIYSF----------GVIPRSDYEI----V   207
            *    .:     .                 .               ..  *

                           D254                                 F286 H290
HadA        DDKVEHPLAAQGDELDGMTVFENVFIPWSHVFHIGNPNH----A-KLYPQRVFD-WLHYH   292
ReTcpA      EDPIEHPLASQGDELDGMTVFDNVFIPWSHVFHLGNPEH----A-KLYPQRVFD-WLHYH   292
CnTcpA      DDPIEHPLASQGDELDGMTVFENVFIPWSHVFHLGNPEH----A-KLYPQRVFD-WVHYH   292
TftD        NDPAEHPLASQGDELDSTTVFDNVFIPWEQVFHIGNPEH----A-KLYPQRIFD-WVHYH   291
CphCI       ADPYDRPLATLGDELDGMVYFDDVLITWDQVQHVGNPEH----A-KWYPQRQFD-WVHLE   294
DcmB1       GSPFDYPLSSRMDENDTIFVFDKVLVPWENVFMYGDVDR----INAFFPQSGFLPRFTFQ   328
HnpA        DDRIEHPLASQGDELDGMTVFDNVFIPWSHVFHLGNPEH----A-KLYPQRVFD-WLHYH   292
NpsA1       ADPYDHPLSTIGDELDGMAYFDDVFIPWENVQHIGNPDH----A-KWYPQRQFD-WVHIE   295
NpcA        ADPYDHPLSTIGDELDGMAYFDDVFIPWENVQHIGNPDH----A-KWYPQRQFD-WVHIE   295
NpdA2       ADPYDRPLATLGDELDGMVYFDNVLIPWAQVQHVGNPDH----A-KWYPQRQFD-WVHLE   294
PheA1       GSPFDYPLSSRFDENDAVIVLDNVFIPWENVFAYKNVKV----VNGFFVETGFVNRFTFQ   294
TtHpaB      DSPFDHPLSSRVEEMDCLVIFDDVLVPWERVFILGNVEL----CNNAYAATGALNHMAHQ   287
EcHpaB      GSPYDYPLSSRFDENDAILVMDNVLIPWENVLIYRDFDR----CRRWTMEGGFARMYPLQ   304
C2          DNWYAQAIKSSG---SKMLKLVNVFIPEYRISKAKDMMEGKSAGFGLYPDSKIF-YTPYR   263
             .     : :     .    : .*::   .:    :                       .

HadA        ALIRQMVRAELVAGLAVLITEHIGTNKIPAVQTRVAKLIGFH--------------QAML   338
ReTcpA      ALIRQSVRAELMAGLAILITEHIGTNKIPAVQTRVAKLIGFH--------------QAML   338
CnTcpA      ALIRQSVRAELMAGLAILITEHIGTNKIPAVQTRVAKLIGFH--------------QAML   338
TftD        ILIRQVLRAELIVGLAILITEHIGTSKLPTVSARVAKLVAFH--------------LAMQ   337
CphCI       TQIRQTVHAELMVGLGLLITQALGTSKNPVVQSQLAELIRFR--------------ETCR   340
DcmB1       GCTRLAVKLDFIAGLLMKALEATGAGGFRGVQTRVGEVIGWR--------------NLFW   374
HnpA        ALIRQSVRAELMAGLAILITEHIGTNKIPAVQTRVAKLIGFH--------------QAML   338
NpsA1       TQIRHAVHAELIVGLALLLTNALGTNNNPIVQSQLADLVRFR--------------ETCK   341
NpcA        TQIRHAVHAELIVGLALLLTNALGTNNNPIVQSQLADLVRFR--------------ETCK   341
NpdA2       TQIRQTVHAELMVGLGLLITQSLGTSKNPVVQSQLAELIRFR--------------ETCR   340
PheA1       GCTRFAVKLDFMVGLLMKATEAAGTKQFRGVQANIGEVVAFR--------------NMFW   340
TtHpaB      VVALKTAKTEAFLGVAALMAEGIGADVYGHVQEKIAEIIVYL--------------EAMR   333
EcHpaB      ACVRLAVKLDFITALLKKSLECTGTLEFRGVQADLGEVVAWR--------------NTFW   350
C2          PYFAS-GFSAVSLGIAERMIEAFKEKQRNRVRAYTGANVGLATPALMRIAESTHQVAAAR   322
                         .:     :         *    .  :

HadA        AHLIASEELGFHTPGGHYKPNILIYDFGRALYLENFS---QMIYELVDLSGRSALIFASE   395
ReTcpA      AHIVASEELGFHTPGGAYKPNILIYDFGRALYLENFS---QMIYELVDLSGRSALIFASE   395
CnTcpA      AHIVASEELGFHTPGGAYKPNILIYDFGRALYLENFS---EMIYDLVDLSGRSALIFASE   395
TftD        AHLIASEETGFHTKGGRYKPNPLIYDFGRAHFLQNQM---SVMYELLDLAGRSSLMIPSE   394
CphCI       AFMIAAEETGFHTPGGLYKPNNIFIDFGRAHYLEHQH---EFVNMLIEFCGRGIVIQPTK   397
DcmB1       SLTESMARDPEQWVGDSVIPKLEYGLTYRMFMIQGYP---RIKEIIEQDVASGLIYLPSS   431
HnpA        AHIVASEELGFHTPGGAYKPNILIYDFGRALYLENFS---QMIYELVDLSGRSALIFASE   395
NpsA1       AFAIAAEETGFTTAGGLFKPNNIYVDLGRAHYLENIH---NAVNQLIEFCGRGVVMSPTK   398
NpcA        AFAIAAEETGFTTAGGLFKPNNIYVDLGRAHYLENIH---NAVNQLIEFCGRGVVMSPTK   398
NpdA2       AFMIAAEETGFHTPGGLYKPNNIFIDFGRAHYLEHQH---EMVNMLIEFCGRGIVIQPTA   397
PheA1       ALSTAMAANCE-MRNGVALPNLQYGAAYRVLAPMVWP---RVKQIFEQVVAGGLIQLPSS   396
TtHpaB      AFWTRAEEEAKENAYGLLVPDRGALDGARNLYPRLYP---RIREILEQIGASGLITLPSE   390
EcHpaB      ALSDSMCSEATPWVNGAYLPDHAALQTYRVLAPMAYA---KIKNIIERNVTSGLIYLPSS   407
C2          ALLEKTWEDHRIHGLNHQYPNKETLAFWRTNQAYAVKMCIEAVDRLMAAAGATSFMD---   379
            :              .   *.       *                :        .
```

```
                                                                      R439 F441
HadA            -DQWNDDKLNGWFERMNNGP-VGRPHDRVKIGRVIRDLFLTDWGSRLFVFENFNGTPLQG    453
ReTcpA          -DQWNDEALNGWFERMNNGP-VGQPHDRVKIGRVIRDLFLTDWGNRLFVFENFNGTPLQA    453
CnTcpA          -DQWNDKTLHGWFERMNNGP-VGKPHDRVKIGRVIRDLFLTDWGNRLFVFENFNGTPLQT    453
TftD            -GQWDDSQSGQWFVKLNNGP-KGNPRERVQIGRVIRDLYLTDWGGRQFMFENFNGTPLFA    452
CphCI           -RELDHPYIGPKLQEALRGS-EISARDRIKIFRQISERFLTEFGSRHEMFEKFNGTPPYL    455
DcmB1           SLDFKSPDVRPYLDKYVRGSDGITAVDRVKVMKALWDSIGTEFGGRHELYERNYSGNHEN    491
HnpA            -DQWNDEALNGWFERMNNGP-VGKPHDRVKIGRVIRDLFLTDWGNRLFVFENFNGTPLQA    453
NpsA1           -ADFDHPFLGPKLEEALRGT-SISARDRVSIFRQISERYLTQWGARHEMFEKFNGTPLYL    456
NpcA            -ADFDHPFLGPKLEEALRGT-SISARDRVSIFRQISERYLTQWGARHEMFEKFNGTPLYL    456
NpdA2           -RELDHPYIGPKLQEALRGS-EISARDRIKIFRQISERFLTEFGTRHEMFEKFNGTPPYL    455
PheA1           ANDFLNPELRPYLDRYYRGS-GIGAEERVKLMKMIWDAIGTEFGGRHELYEINYAGNHEN    455
TtHpaB          -KDFKGP-LGPFLEKFLQGA-ALEAKERVALFRLAWDMTLSGFGARQELYERFFFGDPVR    447
EcHpaB          ARDLNNPQIDQYLAKYVRGSNGMDHVQRIKILKLMWDAIGSEFGGRHELYEINYSGSQDE    467
C2              ---------------------------NSELQRLFRDAHMTGAHA----YTDY------D    402
                                           .  : :   :   :        :

                   L457
HadA            IRMLTMQRAEFSGSGP-YGKLARQVCGIDSAVTDDTE---YRKTA----------DYAKA    499
ReTcpA          IRMLTMQRAEFSAAGP-YGTLARKVCGIELTEGHDSE---YKATA----------GYAQA    499
CnTcpA          IRMLTMQRAEFSAAGP-YCTLARKVCGIELTEVHESE---YKATA----------GYAQA    499
TftD            VFAATMTRDDMSAAGT-YGKFASQVCGIEFGGAEPTA---YAATA----------DYAKA    498
CphCI           INILTMQRTEYQVDGP-LTQLARDVLGFGDTAELGRRAEEAEKASHYASVK-YQPEYARS    513
DcmB1           VKAELLFAAQNRGHASSMKGLAEQCL------------SEYDLDG-WTVPDLIGNDDVSF    538
HnpA            IRMLTMQRAEFSAAGP-YGTLARKVCGIELGDDHESE---YKATA----------GYAQA    499
NpsA1           VRLLTMQRTEYQVDGP-LTDLARQVLGFGDTEALAARAAEVEKNSNWASVA-YQPEYARE    514
NpcA            VRLLTMQRTEYQVDGP-LTDLARQVLGFGDTEALAARAAEVEKNSNWASVA-YQPEYARE    514
NpdA2           INILTMQRTEYQVDGP-LTQLARDVLGFGDTAELGKRAEEADKASHYASVK-FQPEYARS    513
PheA1           IRLETLKIADVTGDSERFKAFVDSAL------------DDYDLHG-WVNDTWVSPKKEIV    502
TtHpaB          MYQTLYNVYNKEPYKERIRAFLKESL------------KVFEEVQ-A-------------    481
EcHpaB          IRLQCLRQAQSSGNMDKMMAMVDRCL------------SEYDQNG-WTVPHLHNNDDINM    514
C2              VCA---------------QILGRELMGMEPDPTMV-------------------------    422
                :                   :

HadA            LDAAR--HQEEVALAGAMAI--    517
ReTcpA          LDSAR--HQEKLALSGTMTV--    517
CnTcpA          LDSAR--HQEKLALSGTMTV--    517
TftD            LDKGL--APEPAAAESATS---    515
CphCI           QDVHDGYIENAESSDPAVTTPA    535
DcmB1           FKNR------------------    542
HnpA            LDSAR--HQEKLALSGTMTV--    517
NpsA1           QDVRDGYYKETEKV--------    528
NpcA            QDVRDGYYKETEKV--------    528
NpdA2           QDVHDGYIDDAEESDTAVTTPA    535
PheA1           QK--------------------    504
TtHpaB          ----------------------    481
EcHpaB          LDKLL-------K---------    520
C2              ----------------------    422
```

Figure S4. Full length amino acid sequence alignment of Group D two-component monooxygenases. Multiple amino acid sequence alignment was performed using ClustalW (EMBL-EBI). HadA monooxygenase from *R. pickettii* (this study), *Re*TcpA from *R. eutropha* JMP134 (89% amino acid sequence identity with HadA), *Cn*TcpA from *C. nantongensis* X1^T (86% amino acid sequence identity with HadA), TftD from *B. cepacia* AC1100 (64% amino acid sequence identity with HadA), CphC-I from *A. chlorophenolicus* A6 (42% amino acid sequence identity with HadA), DcmB1 from *Rhodococcus* sp. JT-3 (19% amino acid sequence identity with HadA), HnpA from *Cupriavidus* sp. CNP-8 (87% amino acid sequence identity with HadA), NpsA1 from *Rhodococcus sp.* strain PN1 (43% amino acid sequence identity with HadA), NpcA from *R. opacus* SAO101 (43% amino acid sequence identity with HadA), NpdA2 from *Arthrobacter sp.* Strain JS443 (42% amino acid sequence identity with HadA), PheA1 from *B. thermoglucosidasius* A7 (24% amino acid sequence identity with HadA), *Tt*HpaB from *T. thermophilus* HB8 (25% amino acid sequence identity with HadA), and *Ec*HpaB from *E. coli* (19% amino acid sequence identity with HadA). The FMNH$^-$-dependent monooxygenase, *p*-hydroxyphenylacetate hydroxylase (C_2) from *A. baumannii* (14% amino acid sequence identity with HadA), is also included. Yellow, green, and cyan highlight residues involved in FADH$^-$ binding, 4NP binding in the active site, and 4NP binding at the subunit interface, respectively.

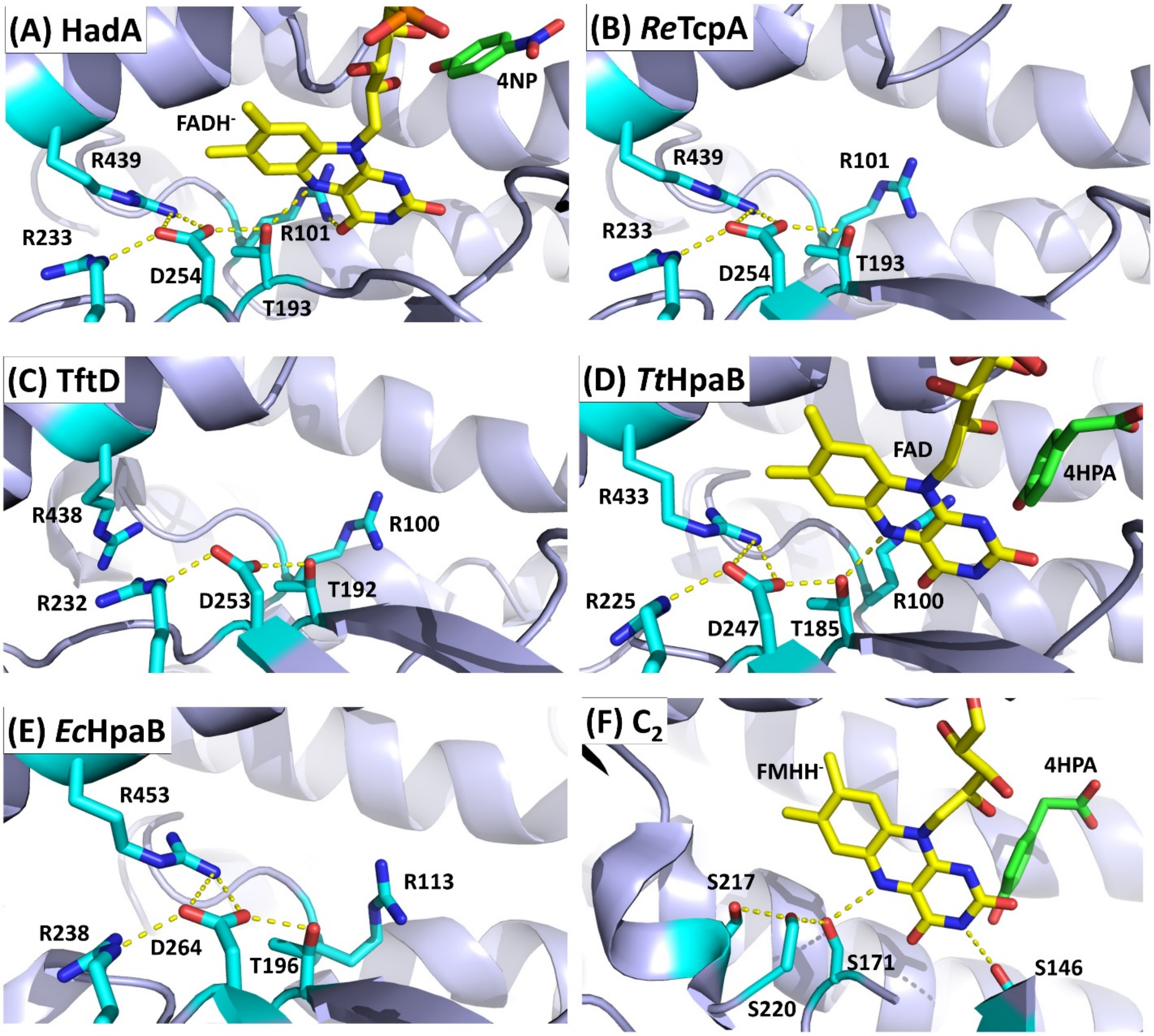

Figure S5. Comparison of the isoalloxazine binding pocket in HadA and its homologs. Conserved environment surrounding the isoalloxazine moiety of the flavin in Group D flavin-dependent monooxygenases including (A) HadA monooxygenase from *R. pickettii*, (B) *Re*TcpA from *R. eutropha* JMP134 (pdb code: 4G5E), (C) TftD from *B. cepacia* AC1100 (pdb code: 3HWC), (D) *Tt*HpaB from *T. thermophilus* HB8 (pdb code: 2YYJ), (E) *Ec*HpaB from *E. coli* (pdb code: 6EB0) and (F) C_2 from *A. baumannii* (pdb code: 2JBT) are illustrated.

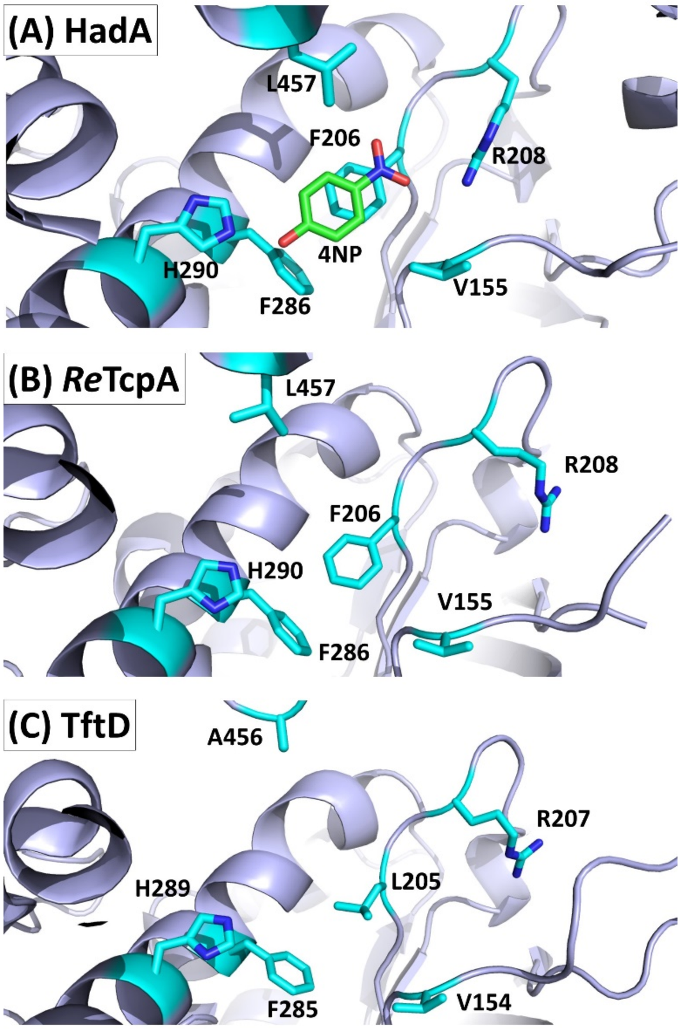

Figure S6. Comparison of phenol substrate binding pockets in dehalogenating flavin-dependent enzymes. The conserved histidine residue catalyzing electrophilic aromatic substitution in the active sites of (A) HadA monooxygenase from *R. pickettii*, (B) *Re*TcpA from *R. eutropha* JMP134 (pdb code: 4G5E), and (C) TftD from *B. cepacia* AC1100 (pdb code: 3HWC) is mostly surrounded by hydrophobic residues.

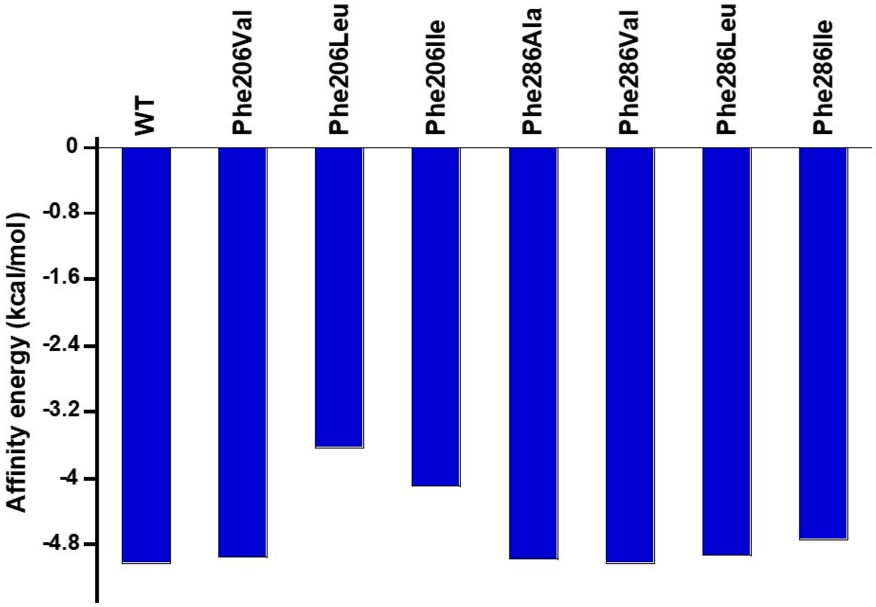

Figure S7. Bar graph shows affinity energy resulting from the binding of 4NP at the active site of $HadA_{Phe206}$ and $HadA_{Phe286}$ variants calculated by molecular docking using the AutoDock Vina software.

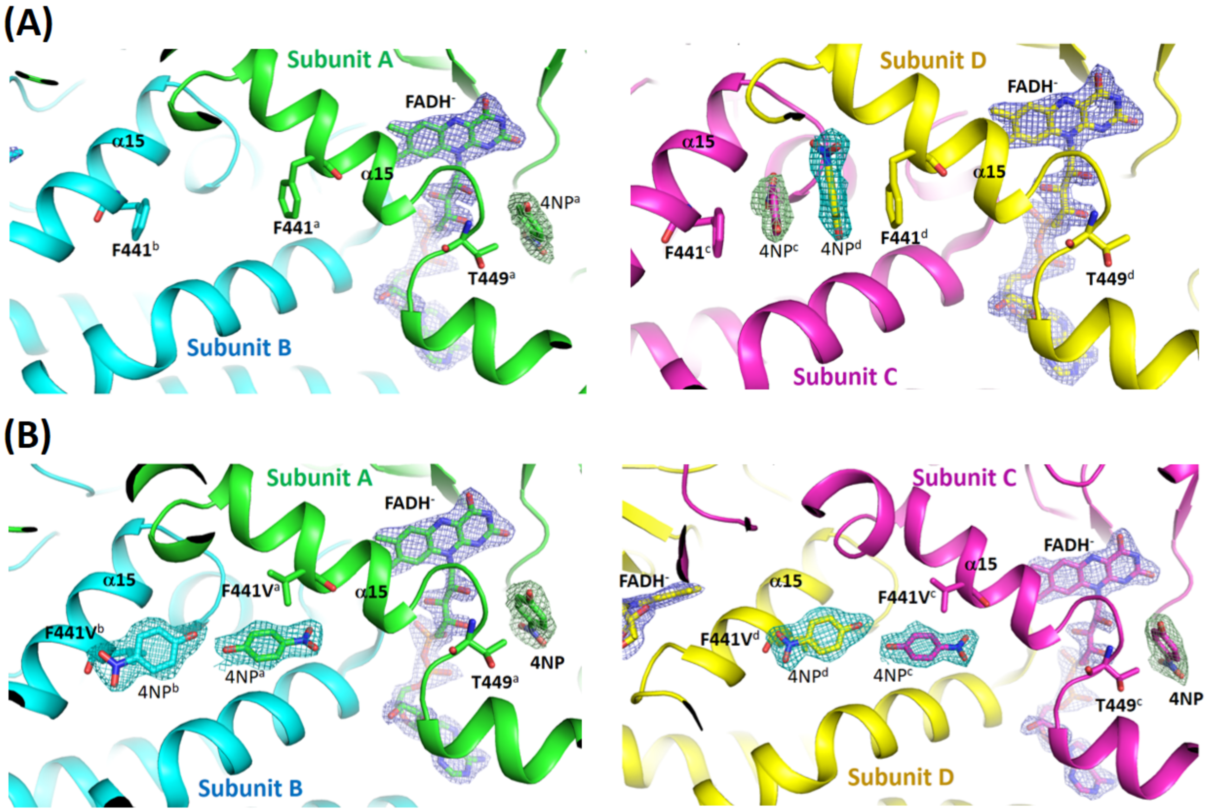

Figure S8. Electron density map of ligands in the HadA structures. (A) $HadA_{WT}$: we noticed that 4NP is only found in one active site of the $HadA_{WT}$ dimer which does not contain a quadruple π-stacking feature at the dimerization interface. (B) $HadA_{Phe441Val}$ variant: two 4NP molecules are bound with van der Waals interactions at helix α15 of the dimerization interface of each dimer. It should be noted that 4NP is observed at the active site of subunits A and C of each dimer. The 2fofc-map density is contoured at 2σ and 1σ for $FADH^{-}$ and 4NP, respectively.

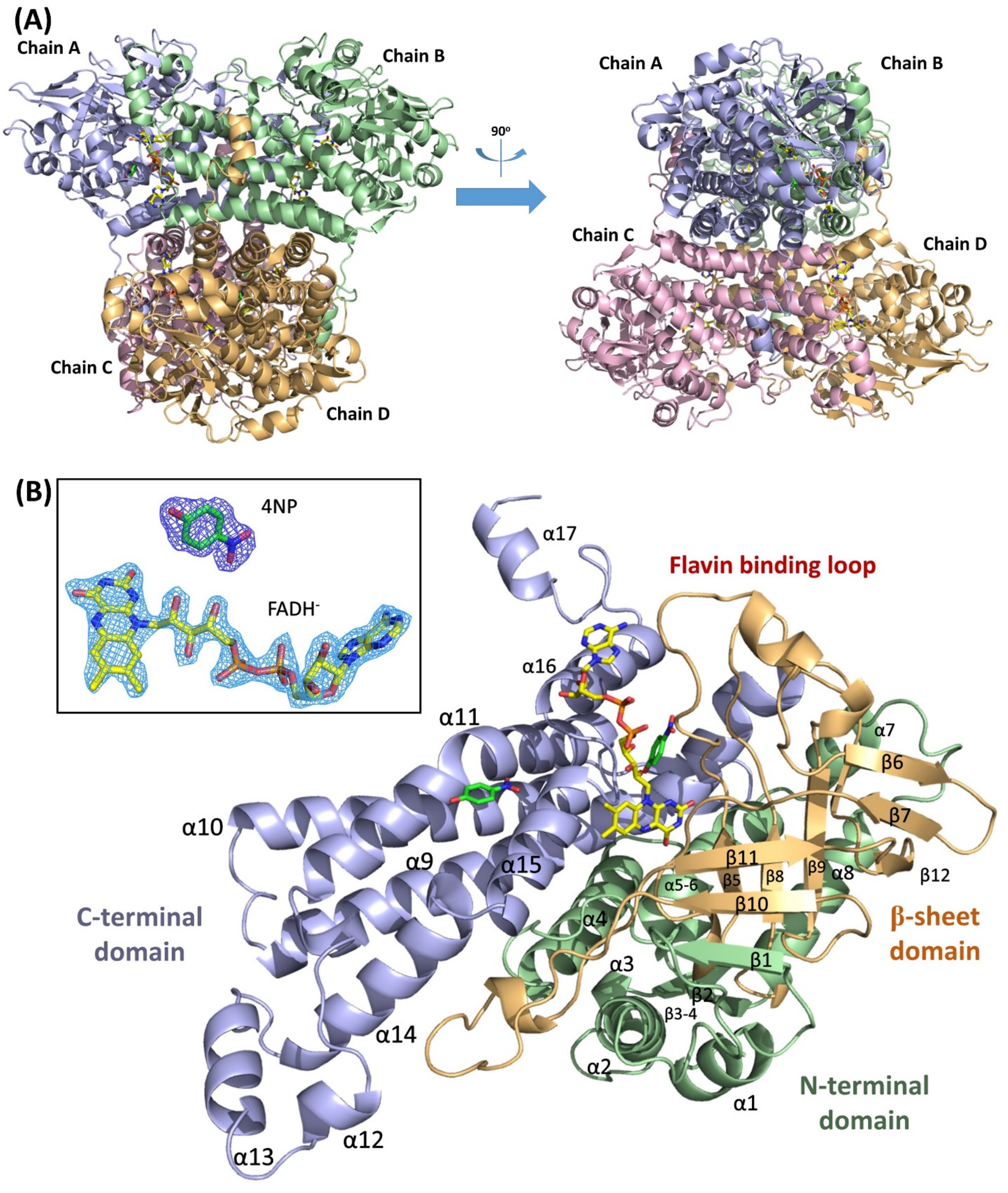

Figure S9. The crystal structure of HadA$_{Phe441Val}$-FADH^{-}-4NP complex. (A) A tetrameric quaternary structure of HadA$_{Phe441Val}$ co-complexed with FADH^{-} and 4NP. (B) Similar to the structure of HadA$_{WT}$, three domains of Subunit A include the N-terminal domain (green part), the β-sheet domain (orange part), and C-terminal domain (purple part). FADH^{-} is shown in yellow while 4NP is shown in green. *Inset* in (B) is an electron density map of FADH^{-} and 4NP in the active site.

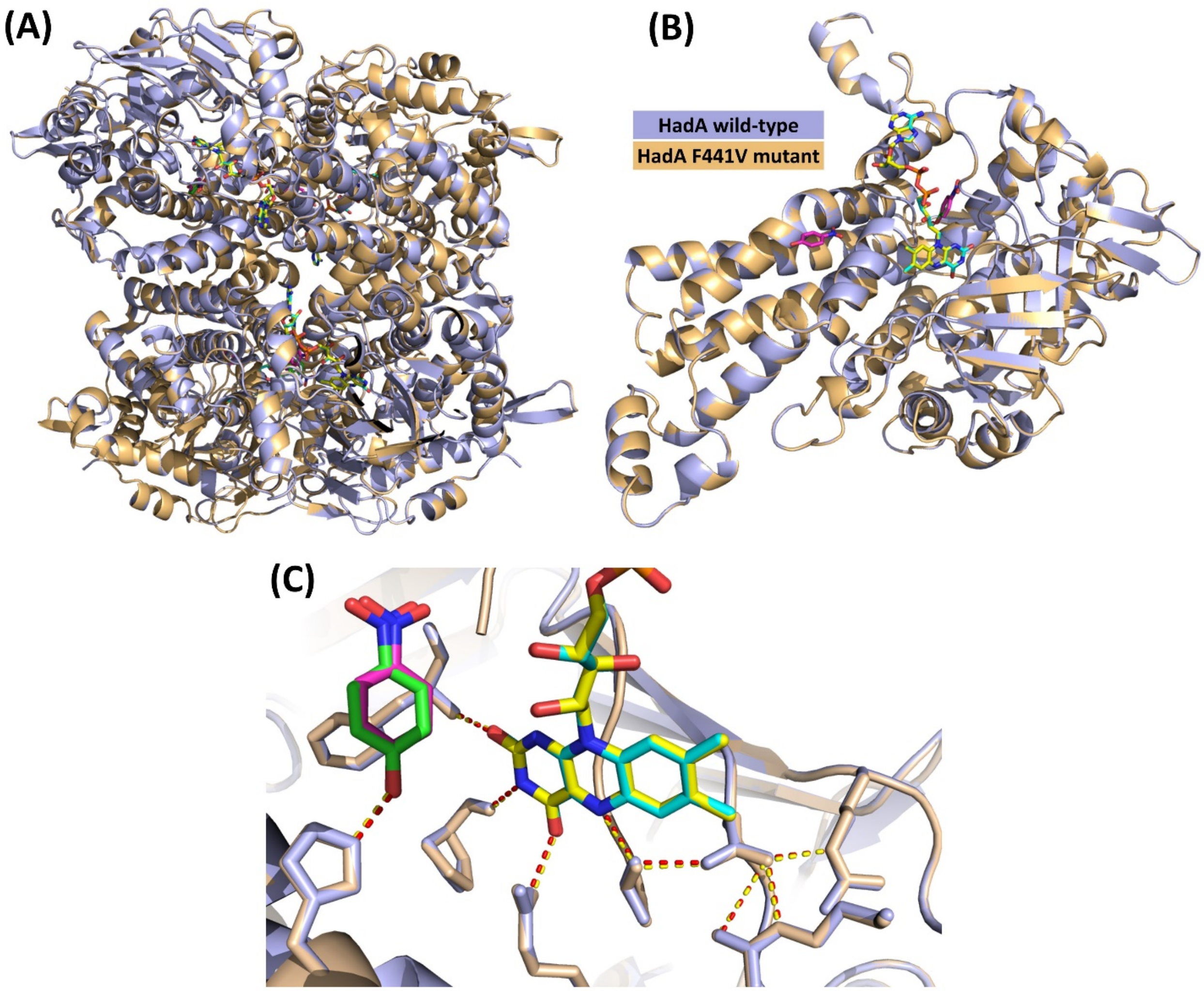

Figure S10. Comparison of structures of $HadA_{WT}$-$FADH^{-}$-4NP and $HadA_{Phe441Val}$-$FADH^{-}$-4NP complexes. (A) A tetrameric structure of the $HadA_{Phe441Val}$-$FADH^{-}$-4NP complex (orange structure) is superimposed with the structure of $HadA_{WT}$-$FADH^{-}$-4NP (purple structure). (B) Superposition of Subunit A of the $HadA_{Phe441Val}$-$FADH^{-}$-4NP complex (orange structure) with the structure of $HadA_{WT}$-$FADH^{-}$-4NP (purple structure) with RMSD of 0.143. (C) Similar active site regions of $HadA_{WT}$ and $HadA_{Phe441Val}$. $FADH^{-}$ and 4NP from $HadA_{WT}$ are shown in yellow and green in the structures respectively, while $FADH^{-}$ and 4NP of $HadA_{Phe441Val}$ are shown in cyan and magenta, respectively.

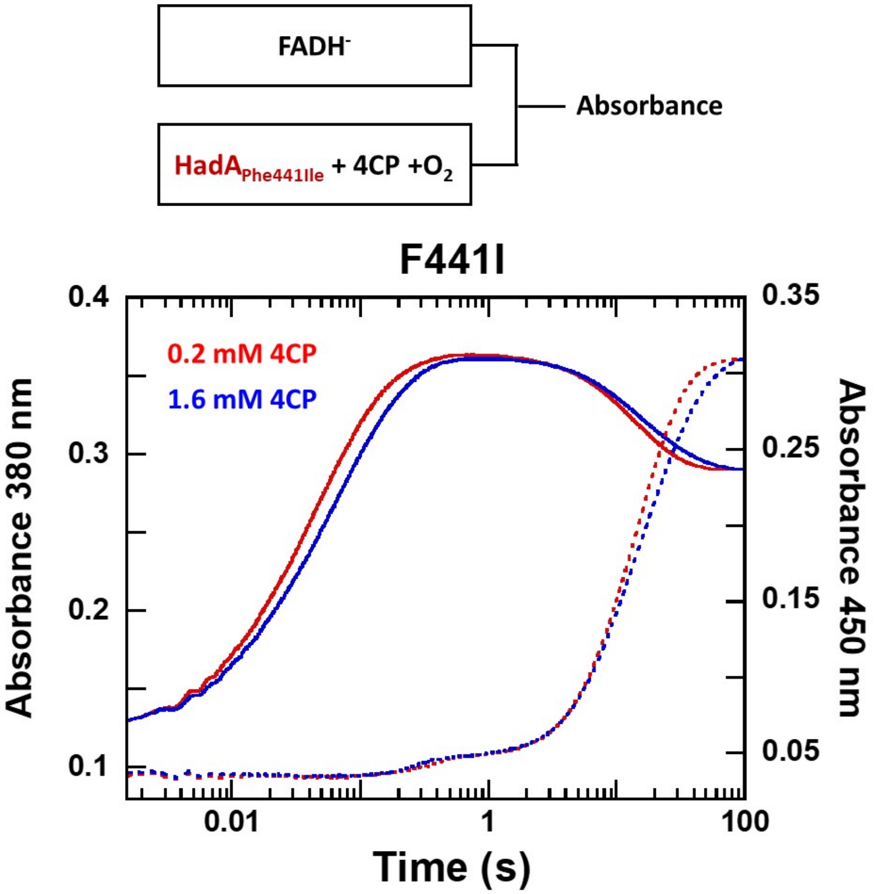

Figure S11. Absence of dead-end complex formation in the HadA$_{Phe441Ile}$ variant. An anaerobic solution of FADH$^-$ (25 µM) was rapidly mixed with an air saturated HadA$_{Phe441Ile}$ variant (75 µM) that was pre-incubated with 0.2 mM 4CP (red lines) or 1.6 mM 4CP (blue lines) in 20 mM HEPES pH 7.5. Absorption changes at the wavelengths 380 nm (solid line) and 450 nm (dashed line) were monitored to detect formation of C4a-hydroperoxy-FAD intermediate and oxidized FAD, respectively. No substrate inhibition was observed in HadA$_{Phe441Ile}$.

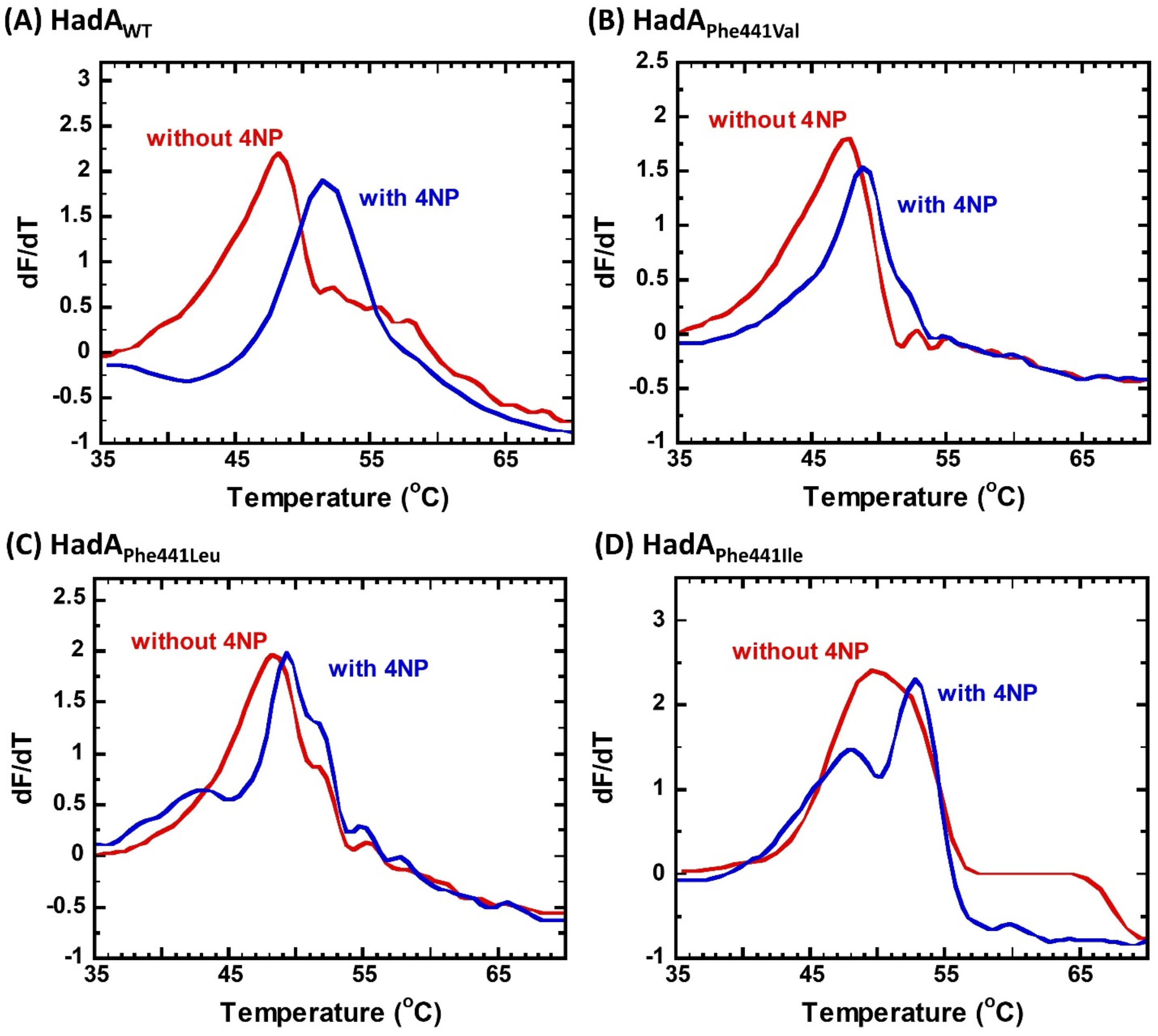

Figure S12. Melting curves of HadA$_{WT}$ and HadA$_{Phe441}$ variants. The melting curves of (A) HadA$_{WT}$, (B) HadA$_{Phe441Val}$, (C) HadA$_{Phe441Leu}$, and (D) HadA$_{Phe441Ile}$ are presented by plotting the first derivative of the fluorescence emission as a function of temperature. Red and blue lines represent the melting curves of apo-enzyme and HadA-4NP complexes, respectively.

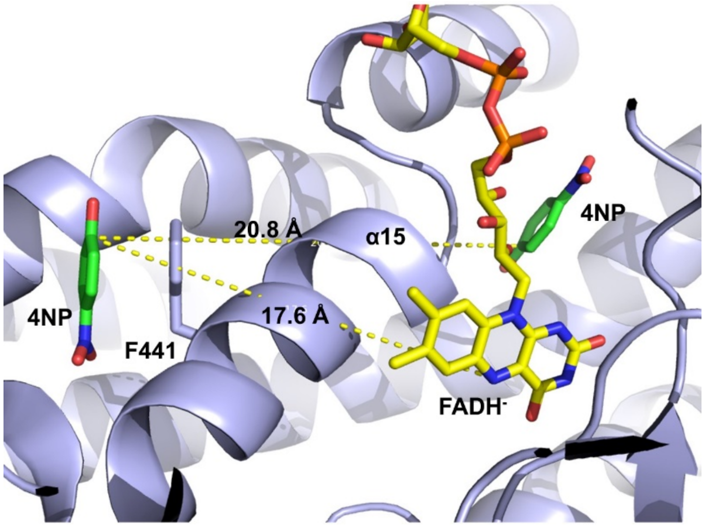

Figure S13. Distance between 4NP bound at the subunit interface and at the active site near $FADH^-$. $FADH^-$ (yellow molecule) and 4NP (green molecule) bind at the active site, while another molecule of 4NP binds at the subunit interface. These two binding pockets are separated by the α15 helix.

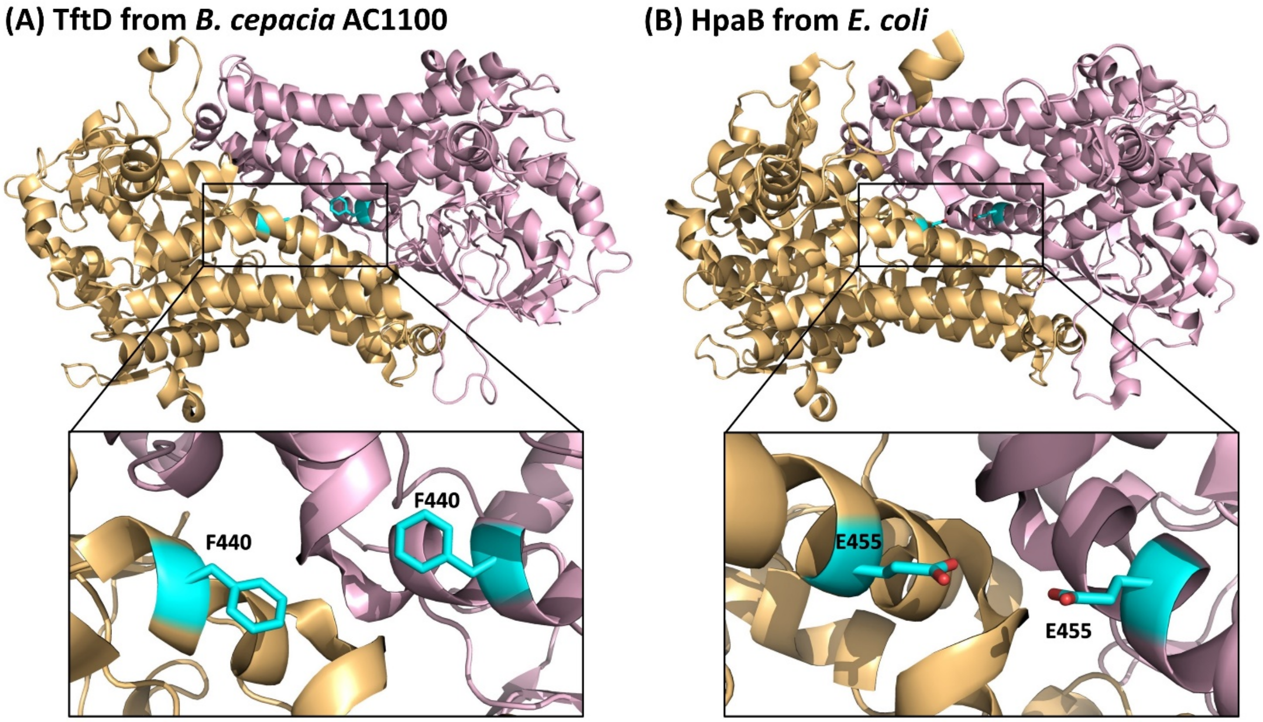

Figure S14. Analysis of the environment around the subunit interface of HadA's homologs. (A) The subunit interface of TftD from *B. cepacia* AC1100 (pdb code: 3HWC, 64% amino acid sequence identity with HadA) reveals the conserved Phe440 residue which may promote aromatic substrate binding and cause dead-end complex formation. (B) The subunit interface of *Ec*HpaB from *E. coli* (pdb code: 6EB0, 19% amino acid sequence identity with HadA) contains Glu455, which is a more hydrophilic environment than found for the dehalogenating flavin-dependent enzymes.

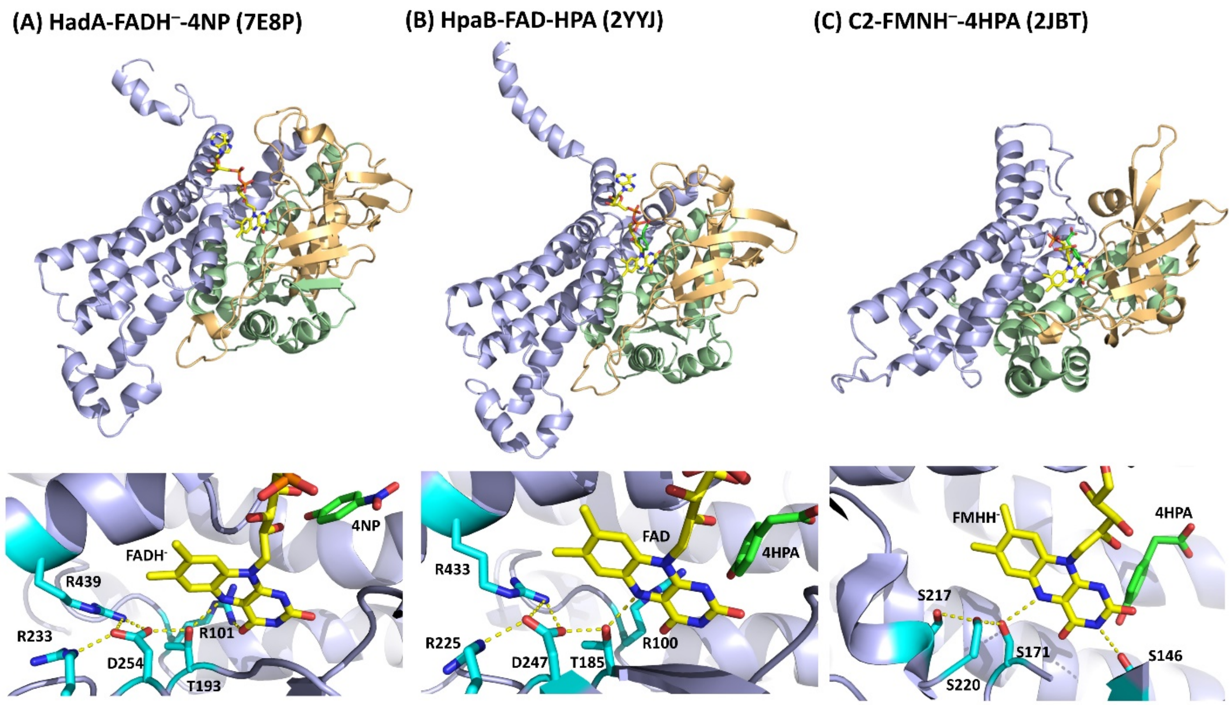

Figure S15. Comparison of the overall and active site structures of HadA and other enzymes of the Class D flavin-dependent monooxygenases. (A) Structures of HadA-FADH$^-$-4NP (this work), (B) *Tt*HpaB-FAD-4HPA from *T. thermophilus* HB8 (pdb code: 2YYJ) and (C) C_2-FMNH$^-$-4HPA from *A. baumannii* (pdb code: 2JBT) co-complexed with flavin and an aromatic substrate.
